# Tumor stemness score to estimate epithelial-to-mesenchymal transition (EMT) and cancer stem cells (CSCs) characterization and to predict the prognosis and immunotherapy response in bladder urothelial carcinoma

**DOI:** 10.1186/s13287-023-03239-1

**Published:** 2023-02-01

**Authors:** Yanlong Zhang, Xin Zhang, Xuefeng Huang, Xiaomeng Tang, Menghan Zhang, Ziyi Li, Xiaopeng Hu, Min Zhang, Xi Wang, Yong Yan

**Affiliations:** 1 Department of Urology, Beijing Shijitan Hospital, Capital Medical University, Beijing, China; 2 Department of Urology, Beijing Chao-Yang Hospital, Capital Medical University, Beijing, China; 3 Institute of Urology, Capital Medical University, Beijing, China; 4 Beijing Ditan Hospital, Capital Medical University, Beijing, China; 5 Beijing Key Laboratory of Emerging Infectious Diseases, Institute of Infectious Diseases, Beijing Ditan Hospital, Capital Medical University, Beijing, 100015 China; 6 Beijing Institute of Infectious Diseases, Beijing, 100015 China; 7 National Center for Infectious Diseases, Beijing Ditan Hospital, Capital Medical University, Beijing, 100015 China; 8 Department of Research Ward, Beijing Chao-Yang Hospital, Capital Medical University, Beijing, 100020 China

**Keywords:** Epithelial-to-mesenchymal transition (EMT), Bladder urothelial carcinoma (BLCA), Cancer stem cells (CSCs), Tumor immunology, Prognostic biomarker, Immunotherapy, Single-cell sequencing

## Abstract

**Background:**

A growing number of investigations have suggested a close link between cancer stem cells (CSCs), epithelial-to-mesenchymal transition (EMT), and the tumor microenvironment (TME). However, the relationships between these physiological processes in bladder urothelial carcinoma (BLCA) remain unclear.

**Methods:**

We first explored biomarkers of tumor stemness (TS) by single-cell sequencing analysis. Then, subtypes of bladder urothelial carcinoma (BLCA) were identified using clustering analysis based on TS biomarkers. The TS score was constructed using principal component analysis to quantify tumor stemness in BLCA. Then, meta-analysis was performed to measure the hazard ratio of the TS score in BLCA cohorts. Moreover, we evaluated the clinical value of the TS score for predicting the response to tumor immunotherapy using immunotherapy cohorts. Finally, we built an EMT cell model by treating T24 cells with TGF-β and validated the relationship between the TS score and the EMT process in tumors by real-time quantitative PCR, cell invasion assays, and RNA-seq. In total, 3846 BLCA cells, 6 cell lines, 1627 BLCA samples, and 9858 samples from 32 other types of tumors were included in our study.

**Results:**

Three TS clusters and two TS-related gene clusters were identified with differential EMT activity status, CSC features, and TME characteristics in BLCA. Then, a TS scoring system was established with 61 TS-related genes to quantify the TS. The prognostic value of the TS score was then confirmed in multiple independent cohorts. A high TS score was associated with high EMT activity, CSC characteristics, high stromal cell content, high TP53 mutation rate, poor prognosis, and high tumor immunotherapy tolerance. The cell line experiment and RNA-seq further validated that our TS score can reflect the EMT and CSC characterization of tumor cells.

**Conclusion:**

Overall, this research provides a better understanding of tumor invasion and metastasis mechanisms through an analysis of TS patterns with different EMT processes and CSC characteristics. The TS score provides an index for EMT and CSC research and helps clinicians develop treatment plans and predict outcomes for patients.

**Supplementary Information:**

The online version contains supplementary material available at 10.1186/s13287-023-03239-1.

## Introduction

**Fig. 1:**
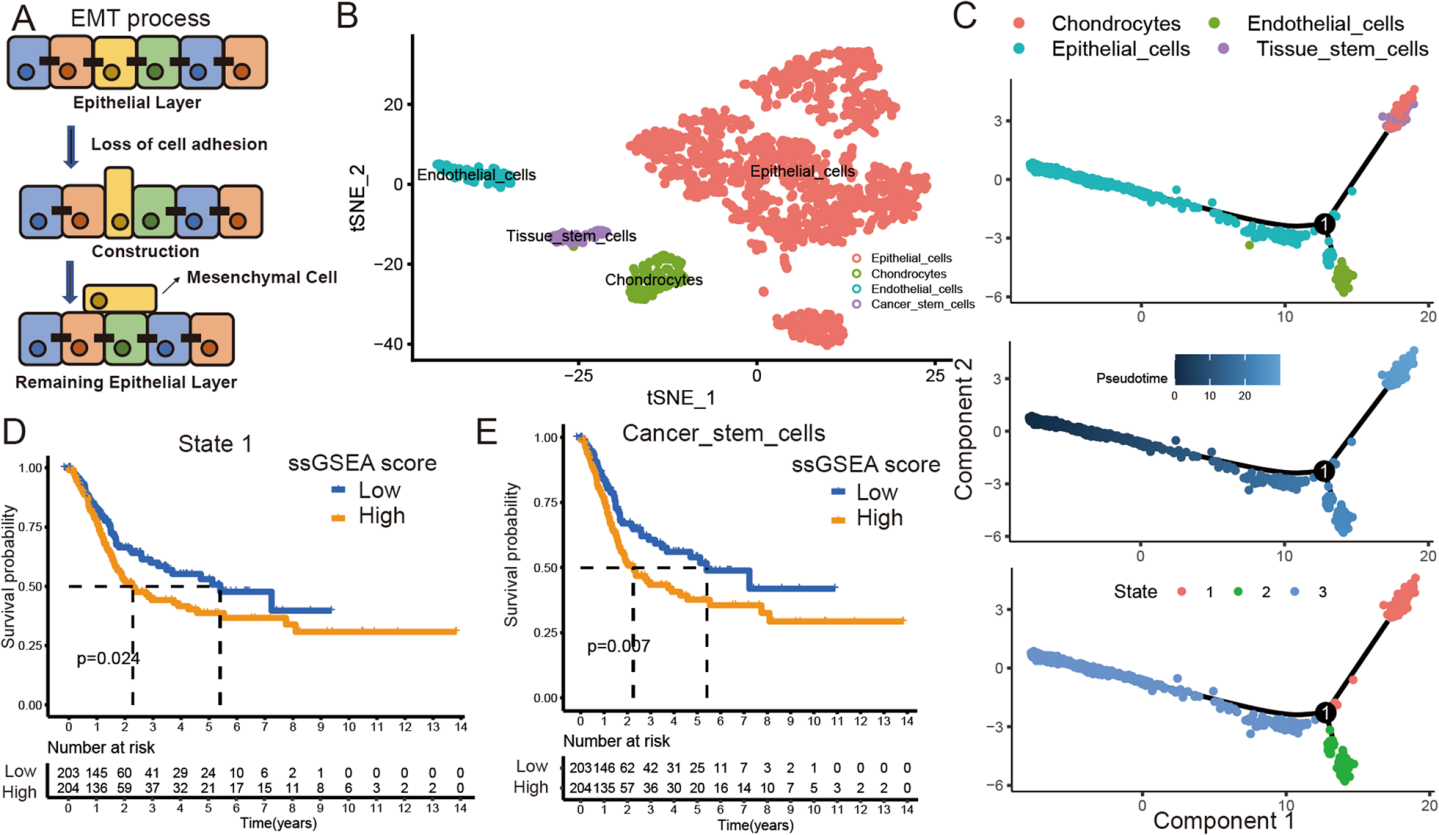
Overview of single-cell and prognostic value of each type cell’s biomarker in BLCA tissue. **A** The EMT process in tumor tissue. **B** The t-SNE plot of all the single cells, with each color coded for 4 major cell types. **C** Differentiation trajectory of epithelial cells in BLCA, with each color coded for cell types (top), pseudotime (medium), and statues (bottom). **D** Kaplan–Meier curves for high/low state 1 ssGSEA score groups in TCGA BLCA cohort. Log-rank test, *p* = 0.024. **E** Kaplan–Meier curves for high/low tissue stem cells ssGSEA score groups in TCGA BLCA cohort. Log-rank test, *p* = 0.007

Epithelial-to-mesenchymal transition (EMT) is the reversible recoding of epithelial cells to mesenchymal cells [1, 2] (Fig. 1A). During EMT in embryonic development, epithelial cells lose all vestiges of their epithelial origin and acquire a fully mesenchymal phenotype in a process known as complete EMT, which is typically characterized by a so-called cadherin switch [3]. Because mesenchymal cells comprise a variety of cell types, epithelial cells can transform into multiple lineages during the EMT process [4]. Cancer stem cells (CSCs) have the features of mesenchymal cells and the principal properties of self-renewal, clonal tumor initiation capacity, and clonal long-term repopulation potential [5]. Many studies have indicated that epithelial tumor cells can transform into CSCs and invade muscle [6, 7]. Although many previous studies have focused on the relationships among EMT, the biological characteristics of tumor cells, and cancer patient prognosis, few researchers have focused on the link between EMT and CSCs. Thus, it is necessary to identify a signature of tumor stemness (TS) to explore the relationship between EMT and CSCs in cancer.

Bladder urothelial carcinoma (BLCA) is one of the most common and deadly urinary system-associated cancers [8]. Although the treatments for BLCA have improved, the recurrence rate remains high [9]. In one study, the ten-year recurrence rate of BLCA in patients with nonmuscle-invasive bladder cancer (NMIBC) was 74%, and these patients ultimately developed muscle-invasive bladder cancer (MIBC) [10]. Since BLCA is an epithelial tumor, many researchers have explored the relationship between EMT and BLCA. Through EMT, epithelial tumor cells transform into mesenchymal cells, and some epithelial tumor cells gain CSC characteristics, exhibiting strong proliferation capacities and invasiveness [11–13]. When these cells exit the bladder muscle layer, patients with NMIBC develop MIBC, necessitating radical cystectomy and leading to a poor prognosis and quality of life [14]. Therefore, we hypothesize that these CSCs from EMT are critical factors in the progression of NMIBC into MIBC and that TS can accurately reflect BLCA prognosis.

Recently, as knowledge of tumor immunology has progressed, immunotherapy options have been developed and applied that have shown significant effects against BLCA [15–17]. However, due to tumor cell heterogeneity and the tumor microenvironment (TME), immunotherapy is only effective for some BLCA patients [18]. The identification of accurate biomarkers is necessary to predict the outcomes of immunotherapy for BLCA. EMT progression and CSCs are influenced by the tumor microenvironment, including tumor stromal cells and immune cells [19]. Therefore, we propose that CSCs from EMT are associated with the TME and TS can predict the effect of immunotherapy on BLCA.

With the development of high-throughput sequencing, it is possible to seek biomarkers in all genomic biological pathways [20, 21]. Due to the emergence and popularization of single-cell sequencing, we can further research cell characteristics and subtypes at the single-cell level [22, 23]. Additionally, the development of algorithms and the application of machine learning in bioinformatics allow developmental and trajectory analyses based on tumor single-cell sequencing data [24, 25]. These technologies are valuable tools for studying CSCs from EMT and quantizing TS.

In this study, we first identified TS biomarkers associated with EMT and CSCs by single-cell sequencing and developmental trajectory analysis. Then, we identified three TS clusters by unsupervised clustering analysis in The Cancer Genome Atlas (TCGA) cohort. To further explore TS characteristics in the whole genome, we selected TS-related genes by differential and clustering analyses. Next, we identified two TS-related gene clusters and found these clusters with disparate CSC and EMT features at the bulk RNA-seq level. Finally, we designed a TS scoring system to quantify CSCs and EMT characteristics. Our independent BLCA dataset validation studies, cell line validation experiments, and pan-cancer analyses indicated that the TS score could reflect CSC, EMT, and TME characteristics and predict patient prognosis and immunotherapy responses in BLCA and other cancers.

## Materials and methods

### Obtaining and processing of data

The workflow for our study was displayed in Additional file 1: Figure S1A. We collected single-cell mRNA sequencing data of BLCA tissue cells from the Gene Expression Omnibus (GEO: https://www.ncbi.nlm.nih.gov/geo/) database (GSE146137). Then, we collected bulk mRNA sequencing data or mRNA microarray data and corresponding clinical information of BLCA cohorts from the TCGA (https://portal.gdc.cancer.gov), ArrayExpress (https://www.ebi.ac.uk/arrayexpress/) (E-MTAB-4321), and GEO databases (GSE13507 and GSE32894). Meanwhile, we collected mRNA microarray data and mRNA sequencing data of the immunotherapy cohorts, including the melanoma PD-1 treatment cohort and BLCA PD-L1 treatment cohort, from the GEO database (GSE78220) and the R package “IMvigor210CoreBiologies” (IMvigor 210 cohort). Next, we collected mRNA sequencing data and mRNA microarray data of cell lines treated with transforming growth factor-β (TGF-β) from the GEO database (GSE98979, GSE17708, GSE101809, GSE124843, and GSE23952). Finally, the somatic mutation variation data and RNA sequencing data of pan-cancer were collected from the University of California Santa Cruz (UCSC) Xena browser (https://xenabrowser.net). The search strategy of the BLCA cohorts was as follows: (1) include “bladder cancer” and dataset types with RNA-seq or microarray data; (2) include more than one hundred BLCA samples with survival data; and (3) include expression information of model genes. Finally, the RNA sequencing data (fragments per kilobase transcript per million mapped reads [FPKM] values) were transformed into transcripts per kilobase million (TPM) values and dealt with the log2(*n* + 1) processing. The clinical information about the TCGA BLCA cohorts was presented in Table S1.

### Pseudotime analysis of BLCA cells

Single-cell analysis was performed by the R package “Seurat” [26, 27]. We first calculated the mitochondrial gene percentage and deleted the mRNA sequencing data with a feature gene content < 50 and a percentage of mitochondrial gene content > 5%. Next, we selected the top 1500 variant genes for principal component analysis (PCA). We then chose the top 15 principal components for t-distributed stochastic neighbor embedding (t-SNE) analysis. The R package “celldex” was used for cell-type annotation, and the R package “monocle” was used for cell trajectory and pseudotime analysis [28, 29]. The R package “Seurat” was used to identify differentially expressed genes between cell clusters, cell types, and cell statuses by the “wilcox” method, and the filter condition was Log |FC|> 1 and adjusted P value < 0.05 [30].

### Unsupervised clustering analysis

To extract the characteristics of mRNA expression data and verify the TS and TS-related gene clusters, we performed unsupervised clustering methods based on TS biomarkers and TS-related genes in the TCGA cohort. The K-means clustering algorithm was applied and repeated 1,000 times using the R package “ConsensusClusterPlus” to ensure the stability of the subtype analysis [31].

### GO and KEGG functional enrichment analysis

The molecular function of TS biomarkers was identified by gene functional enrichment analysis using Gene Ontology (GO) and Kyoto Encyclopedia of Genes and Genomes (KEGG) databases and the R package “clusterProfiler” (adjusted *P* < 0.05).

### Differentially expressed gene analysis

The differentially expressed genes (DEGs) between the three TS clusters with adjusted *p* values < 0.001 were selected using the R package “limma” for further analysis [30].

### Construction of the TS score and nomogram

We further identified 61 TS-related genes from 904 DEGs between the three TS clusters using univariate Cox regression analysis. Then, we performed a principal component analysis (PCA) based on TCGA BLCA data to quantify the TS and generate a TS score. PC1 and PC2 were extracted to calculate the TS score as follows: TS score = Σ(PC2i – PC1i), where i was the expression of 61 TS-related genes. The advantage of this approach was that it concentrated the score on the largest set of highly correlated (or unrelated) gene blocks, while downweighting the contribution of genes that were not tracked by other set members [32, 33].

Univariate Cox regression analysis was used to explore the correlations among clinical variables, TS scores, and overall survival (OS) time of BLCA patients. We built a prognostic risk score model and nomogram based on the TCGA BLCA cohort and multivariate Cox regression analysis. Finally, Kaplan–Meier (K–M) survival curves and time-dependent receiver operating characteristic (ROC) analysis were used to evaluate the prognostic value of the TS score, risk model, and nomogram. *P* values calculated by log-rank tests < 0.05 and an area under the ROC curve (AUC) > 0.60 indicated that the prediction ability of each index was meaningful.

### Molecular function and pathway analysis

The R package “GSVA” was used to calculate the single-sample gene set enrichment (ssGSEA) score for each sample and quantify the activity level of pathways of each cell type [34]. The stem cell gene set used for ssGSEA was collected from previous studies [35]. The infiltration levels of immune cells in BLCA were quantified by using the R package “CIBERSORT” [36]. The R package “ESTIMATE” was used to evaluate the immune cell and stromal cell content (immune and stromal score) of each BLCA sample [37].

### Cell cultures and reagents

The human bladder cancer cell line T24 used in this research was purchased from the Chinese Academy of Sciences Cell Bank (Shanghai, China) and cultured in McCoy’s 5A medium (iCell, China) containing 10% fetal bovine serum (FBS, BI, ISR). The cells were grown at 37 °C and 5% CO_2_. The human TGF beta 1 (TGF-β1) protein was purchased from MedChemExpress (HY-P7118). T24 cells were treated with TGF-β1 (10 ng/ml) for 48 h.

### RNA extraction and RT‒qPCR methods

Total RNA was extracted using a FastPure Cell/Tissue Total RNA Isolation Kit (Vazyme, China), and the concentration was determined. RNA was then used for cDNA synthesis using HiScript III RT SuperMix for qPCR (+ gDNA wiper) Mix (Vazyme, China). Next, qPCR was conducted using AceQ Universal SYBR qPCR Master Mix (Vazyme, China) according to the instructions provided by the manufacturers. The relative expression levels of the target genes were analyzed using the comparative CT method (2^-ΔΔCt^) with GAPDH as the internal reference. The specific primers for the genes were shown in Additional file 13: Table S2.

### Wound filling assay

T24 (1 × 10^6^ cells per well) cells were seeded into 6-well plates. Identical scratches were made in parallel wells 12 h after seeding the cells using a 200 µl pipette tip. Then, we treated cells with 10 ng/ml TGF-β1 in serum-free McCoy’s 5A medium. The cells were observed and photographed at 0, 12, 24, and 36 h after scratching.

### Cell viability assay method

T24 cells were seeded into 96-well plates at a density of 5,000 cells per well. After cell attachment, TGF-β1 was added at concentrations of 5 and 10 ng/ml. Cell viability was measured at 0, 24, and 48 h using a Cell Counting Kit-8 (CCK-8) (Vazyme, China). Then, 100 μL of culture medium containing 10 μL of CCK-8 solution was incubated in each well at 37 °C for 0.5 h. The absorbance was measured at 450 nm using a microplate reader (BioTek, USA).

### Migration and invasion assays

To explore the effects of TGF-β on T24 cells, migration and invasion assays were performed using chambers (8.0-µm pore size) and matrigel precoated chambers, respectively (Corning, USA). T24 cells (5 × 10^4^) treated with TGF-β1 (10 ng/ml) or not were seeded into the upper chamber with 200 μL serum-free McCoy’s 5A medium. The bottom compartment was supplemented with 500 μL medium containing 10% FBS. After incubation for 48 h, the cells were fixed with absolute methanol and stained with 0.1% crystal violet for 20 min. Finally, the number of T24 cells was counted under an inverted microscope.

### RNA sequencing of T24 cells

RNA sequencing (RNA‐seq) was performed by Novogene (Beijing, China). Briefly, T24 cells treated with or without TGF-β1 (10 ng/ml) for 48 h were used to extract total RNA. RNA quality and integrity were assessed using the RNA Nano 6000 Assay Kit of the Bioanalyzer 2100 system (Agilent Technologies, CA, USA). Then, RNA libraries were prepared and sequenced using a 150 bp pairing end protocol by the Illumina NovaSeq 6000. After removing reads containing adapters, reads containing N bases, and low-quality reads from the raw data, at least 70 million clean reads were generated per sample. In addition, the Q20, Q30, and GC contents of the clean data were calculated. Clean reads were aligned to the reference genome using HISAT2 (v2.0.5). FeatureCounts (v1.5.0-p3) was performed to count the read numbers mapped to each gene. These RNA-seq data generated in our study were stored in GEO (GSE215947) database.

### Statistical analysis

An unpaired Student’s *t test* was used to compare two groups with normally distributed variables, while the Mann–Whitney U test was used to compare two groups with nonnormally distributed variables. For comparisons among three groups, one-way analysis of variance and Kruskal–Wallis tests of variance were used for parametric and nonparametric data, respectively. Contingency table variables were analyzed using the Cehi-square test or Fisher’s exact test. Meta-analysis (random-effect model) was used to identify the hazard ratio (HR) value of the TS score in BLCA. Contingency table variables were analyzed by the chi-square test or Fisher’s exact test. Statistical significance was defined as a two-tailed *p* value < 0.05. All statistical analyses were performed using R software (version 4.1.2).

## Results

### The developmental landscape of bladder cancer cells

We first collected BLCA single-cell sequencing data from 3486 CD45(−) BLCA cells from two patients. We identified seven clusters (clusters 0–6) in these BLCA cells through dimension reduction and cluster analysis (Additional file 1: Figure S1B). Further cell annotation analysis indicated that clusters 0–3 were epithelial cells, cluster 4 comprised chondrocytes, cluster 5 constituted endothelial cells, and cluster 6 contained tissue stem cells (Fig. 1B). Then, we performed a developmental trajectory analysis and identified three branches in BLCA cells (states 1–3). States 1 and 2 represented the transformation of epithelial cells to chondrocytes, tissue stem cells, and endothelial cells (Fig. 1C and Additional file 1: Figure S1C, Additional file 14: Table S3). Therefore, we hypothesized that these states represent EMT processes in BLCA and that chondrocytes, tissue stem cells, and endothelial cells are the three developmental directions of EMT progression. Finally, we explored the expression of a stem cell marker (CD44) in these clusters and found that it was significantly increased in cluster 6 (RGS5 + , KRT7−) (Additional file 1: Figure S1D–J). This result further identified cluster 6 as tissue stem cells in BLCA.

### TS was associated with BLCA prognosis

To further explore the role of EMT progression in the progression and development of BLCA, we analyzed the progression in state 1, state 2, and state 3 to verify the relationship between these states and the OS time of BLCA patients. We identified each cluster, cell type, and state biomarker by differential analysis and set the biomarkers with LogFC > 1 as the gene sets for gene set variation analysis (GSVA). First, K–M survival curve analysis (cutoff value = median ssGSEA score) indicated that the ssGSEA score of state 1 was related to the prognosis of patients in the TCGA BLCA cohort (Fig. 1D, Additional file 2: Figure S2A and B). Because state 1 includes two types of cells, tissue stem cells and chondrocytes, we further calculated the ssGSEA score of these cell types independently, and K–M survival curve analysis (cutoff value = median ssGSEA score) suggested that the ssGSEA score of the tissue stem cells was significantly related to the OS time in the TCGA BLCA cohort (Fig. 1E and Additional file 2: Figure S2C), indicating that CSCs from EMT played a critical role in BLCA progression and development. We referred to the genes specifically associated with tissue stem cells as TS biomarkers. We performed a functional enrichment analysis to further confirm the function of the TS biomarkers and found that these biomarkers were mainly associated with epithelial cell proliferation, extracellular matrix binding, adherens junction, and PI3K-Akt signaling pathways (Additional file 2: Figure S2D–G, Additional file 15: Table S4). This result further confirmed that TS biomarkers were related to EMT progression in BLCA.

### The identification of TS clusters in BLCA

**Fig. 2:**
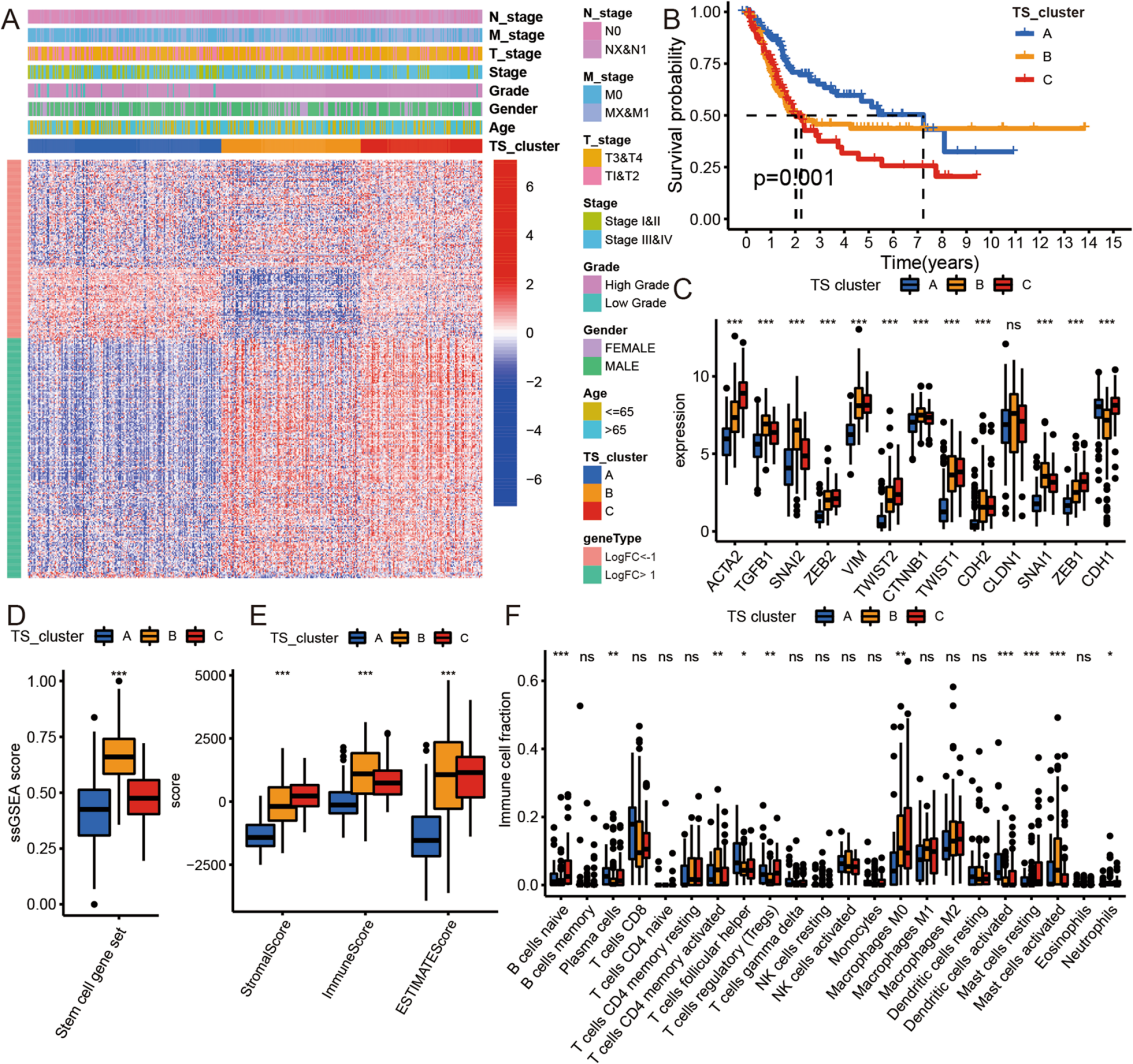
Consensus clustering of TCGA BLCA cohort. **A** Unsupervised clustering of 342 TS biomarkers in the TCGA BLCA cohort. Red represents high expression, and blue represents low expression. LogFC means the log fold change of TS biomarkers from differential analysis with others type cell in single-cell analysis. **B** Kaplan–Meier curves for the three TS clusters of patients. The log-rank test showed an overall *p* = 0.001. **C** Boxplot of 13 EMT biomarkers for three TS clusters in the TCGA BLCA cohort. **D** Boxplot of ssGSEA scores of stem cell gene set for three TS clusters in the TCGA BLCA cohort. **E** Boxplot of stromal score, immune score, and ESTIMATE score for three TS clusters in the TCGA BLCA cohort. **F** Boxplot of 22 immune cells content for three TS clusters in the TCGA BLCA cohort. Kruskal–Wallis test, *p < 0.05, **p < 0.01, ***p < 0.001

Since TS biomarkers could impact BLCA patient prognosis, we further investigated potential TS clusters and characteristics. We first identified three TS clusters in the TCGA BLCA cohort based on unsupervised clustering analysis and the 394 TS markers expression data, namely, TS cluster A (*n* = 175), TS cluster B (*n* = 124), and TS cluster C (*n* = 109) (Fig. 2A and Additional file 3: Fig. S3A–G). Then, PCA displayed a significant separation of the three clusters (Additional file 3: Figure S3H). The K–M survival curve revealed that TS cluster A was related to an obviously better prognosis (Fig. 2B). To further identify the CSC, EMT, and TME characteristics of each cluster, we compared the gene expression of EMT biomarkers, stromal scores, immune scores, ESTIMATE scores, and immune cells among the three TS clusters. We found that the expression of all EMT biomarkers was significantly different among the three TS clusters, except for CLDN1. The expression levels of ACTA2, TGFB1, SNAI2, ZEB2, VIM, TWIST1, TWIST2, CTNNB1, CDH2, CLDN1, SNAI1, and ZEN1 were lower in TS cluster A than in TS clusters B/C, and the expression of CDH1 was lower in TS clusters A/C than in TS cluster B. These results indicated that TS cluster A had lower EMT activity than TS clusters B/C (Fig. 2C). Additionally, we found that TS cluster B had the highest stem cell gene set ssGSEA among the three clusters, indicating that the CSC content was highest in cluster C (Fig. 2D). TS cluster A, in contrast, had the lowest stromal, immune, and ESTIMATE scores among the three clusters. Therefore, cluster A likely has low stromal and immune cell content (Fig. 2E). Finally, we compared the levels of 22 types of immune cells and found differences in naive B cells, plasma cells, memory-activated CD4 T cells, follicular helper T cells, regulatory T cells (Tregs), M0 macrophages, activated dendritic cells, resting mast cells, activated mast cells, and neutrophils; thus, the three TS clusters likely had different TME and immune infiltration characteristics (Fig. 2F).

### Construction of the TS score

**Fig. 3:**
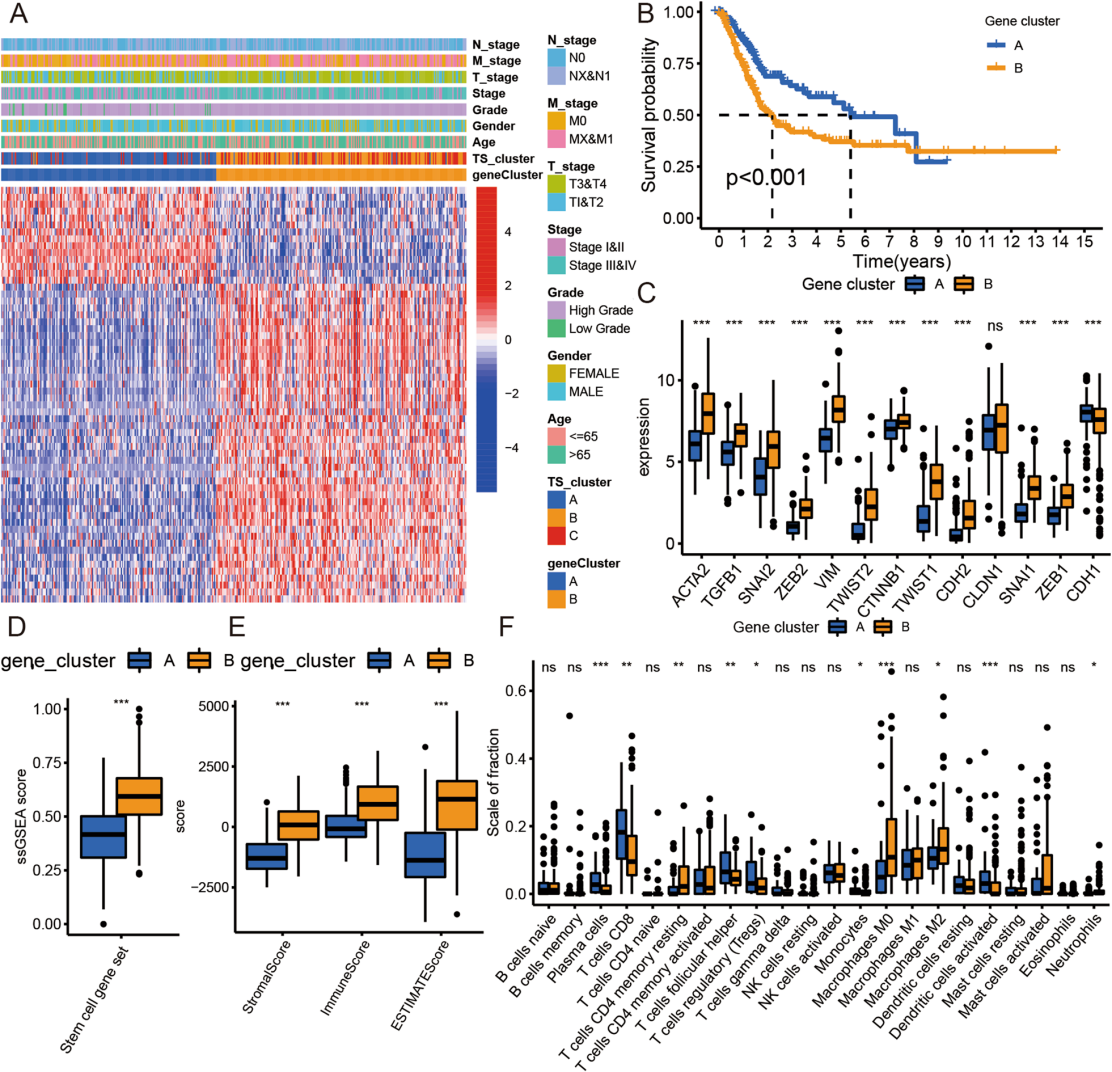
Transcriptomic and metabolic characteristics of the TS-related gene clusters. **A** Unsupervised clustering of 61 TS-related genes in the TCGA BLCA cohort. Red represents high expression, and blue represents low expression. **B** Kaplan–Meier curves for the two TS-related gene clusters of patients. The log-rank test showed an overall *p* < 0.001. **C** Boxplot of 13 EMT biomarkers for two TS-related gene clusters in the TCGA BLCA cohort. **D** Boxplot of ssGSEA scores of stem cell gene set for two TS-related gene clusters in the TCGA BLCA cohort. **E** Boxplot of stromal score, immune score, and ESTIMATE score for two TS-related gene clusters in the TCGA BLCA cohort. **F** Boxplot of 22 immune cell content for two TS-related gene clusters in the TCGA BLCA cohort. Wilcox test, *p < 0.05, **p < 0.01, ***p < 0.001

To identify the biomarkers of each TS cluster, we performed differential expression analysis among the three TS clusters. A total of 904 DEGs were identified in the TCGA BLCA cohort (*p* < 0.001; Additional file 4: Figure S4A, Additional file 16: Table S5). Then, we further identified 61 DEGs related to the OS time of BLCA patients using univariate Cox regression analysis (*p* < 0.001; Additional file 17: Table S6). We classified these DEGs as TS-related genes. We also processed the clustering analysis and confirmed two TS-related gene clusters based on the expression data of these 61 genes, and we found that gene cluster B corresponded to TS clusters B and C (Fig. 3A and Additional file 4: Figure S4B–I).

Moreover, we displayed the OS time of patients from different TS-related gene clusters based on the K–M survival curve. Then, we found that patients from gene cluster B had a shorter OS time than patients from gene cluster A (log-rank test, *p* < 0.001; Fig. 3B). To further confirm the relationship between CSCs, EMT, TME characteristics, and gene clusters, we compared the expression of EMT biomarkers, stromal scores, immune scores, ESTIMATE scores, and immune cell levels between these two gene clusters. We found that ACTA2, TGFB1, SNAI2, ZEB2, VIM, TWIST1, TWIST2, CTNNB1, CDH2, CLDN1, SNAI1, and ZEN1 expression levels were lower in gene cluster A than in gene cluster B, while CDH1 expression was higher in gene cluster A than in gene cluster B (Fig. 3C). These results were the same as those of the TS clustering analysis and further indicated that the EMT activity of gene cluster B was higher than that of gene cluster A. Subsequently, we found that cluster B had a higher stem cell gene set ssGSEA score than that of gene cluster A (Fig. 3D). Moreover, the results indicated that compared with gene cluster B, gene cluster A was associated with lower stromal, immune, and ESTIMATE scores (Fig. 3E). Therefore, gene cluster A, like TS cluster A, likely has low stromal and immune cell content. Finally, we found that the plasma cell, CD8 T cell, resting CD4 memory T cell, follicular helper T cell, Treg, monocyte, M0 macrophage, M2 macrophage, activated dendritic cell, and neutrophil levels differed between the two TS-related gene clusters (Fig. 3F), indicating that these immune cells played an important role in TS progression in BLCA.

**Fig. 4:**
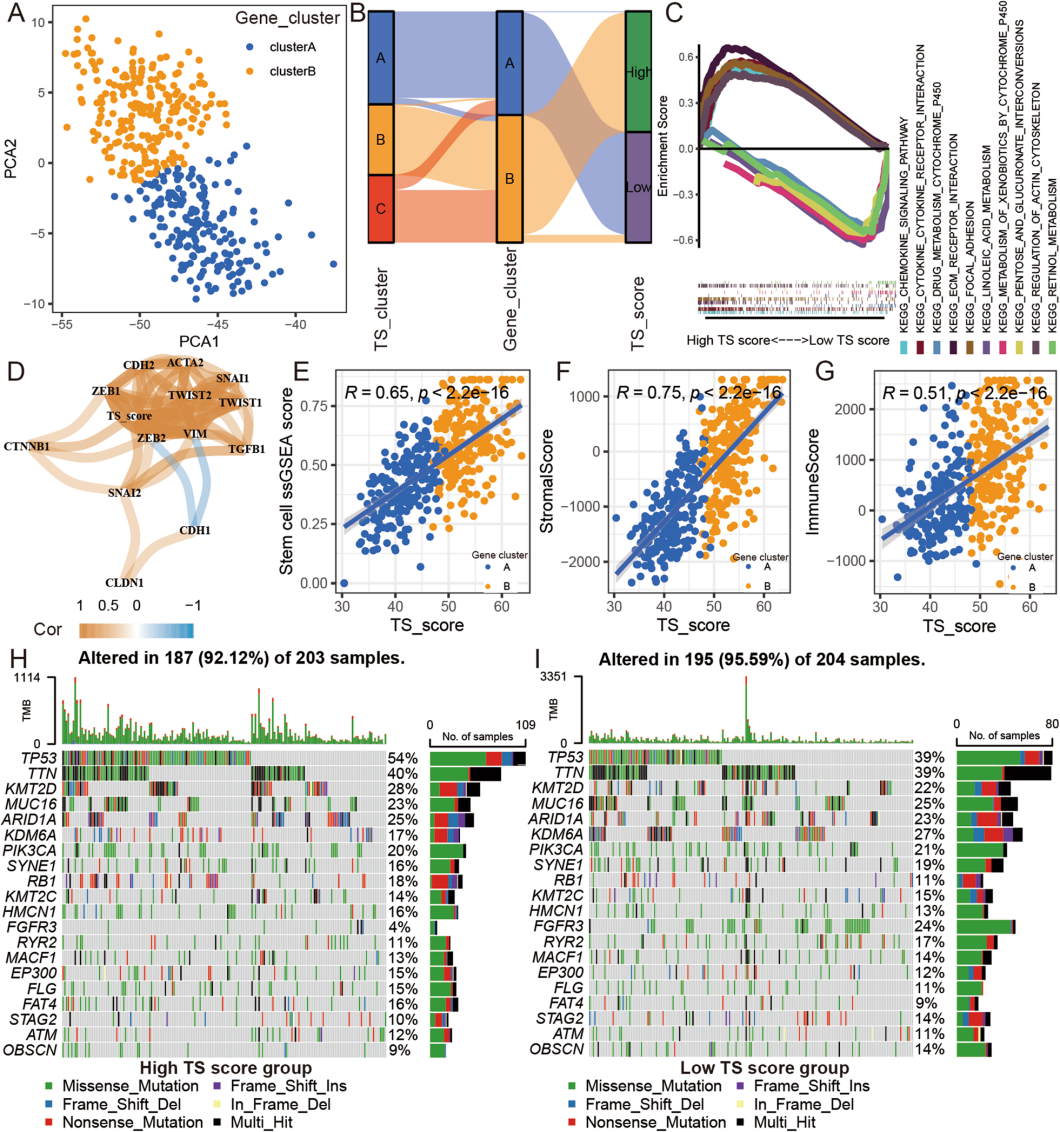
The relationship between TS characteristics and TS score in BLCA. **A** Principal component analysis (PCA) based on 61 TS-related genes expression. **B** Alluvial diagram of TS clusters and TS-related gene distribution in groups with different TS scores. **C** GSEA analysis of high/low TS score group in TCGA BLCA cohort. **D** The correlation network between EMT biomarkers and TS score in TCGA BLCA cohort. Spearman, Cor > 0.3 and *p* < 0.001. **E** The correlation between TS score and ssGSEA score of stem cell gene set in TCGA BLCA cohort. Spearman, Cor = 0.65 and *p* < 0.001. **F** The correlation between TS score and stromal score in TCGA BLCA cohort. Spearman, Cor = 0.75 and *p* < 0.001. **G** The correlation between TS score and immune score in TCGA BLCA cohort. Spearman, Cor = 0.51 and *p* < 0.001. The oncoPrint was constructed using high TS score group (**H**) and low TS score group (**I**). Individual patients are represented in each column in TCGA BLCA cohort

To obtain a quantitative biomarker of TS in BLCA samples, we used the PCA algorithm to calculate the TS score (PCA2 minus PCA1) using the TS-related genes (Fig. 4A and Additional file 18: Table S7). The corresponding relationship of TS clusters and gene clusters was displayed in Fig. 4B.

### The TS score was closely related to CSCs, EMT, TME characteristics, and TP53 mutation in BLCA

We performed GSEA based on KEGG pathways between the high and low TS score groups (cutoff value = 48.50 [median value]) in the TCGA BLCA group to verify the biological functions associated with the TS score. The results indicated that the pathways of focal adhesion, ECM receptor interaction, regulation of actin cytoskeleton, and chemokine signaling pathway were activated in the high TS score group, and pathways of metabolism of xenobiotics by cytochrome P450, linoleic acid metabolism, retinol metabolism, and pentose and glucuronate interconversions were activated in the low TS score group. (Fig. 4C, Additional file 19: Table S8). Then, the correlation analysis indicated that the TS score was closely correlated with the expression of EMT biomarkers (Fig. 4D). We also found a close relationship between the TS score and the ssGSEA score of the stem cell set, stromal score, and immune score (Fig. 4E–G). All these results suggest that the TS score could reflect the EMT level, CSC features, and TME characteristics of BLCA tumors. Finally, we found a significant difference in the expression levels of immune checkpoint genes between the low and high TS score groups. (Additional file 5: Figure S5A). Moreover, we found that plasma cell, CD8 T cell, follicular helper T cell, Treg, and dendritic cell contents were higher in the low TS score group, while resting memory CD4 T cell, M0 macrophage, M2 macrophage, and neutrophil contents were higher in the high TS score group (Additional file 5: Figure S5B).

To further explore the driving factor of TS progression in BLCA, we analyzed DNA mutations and found no significant relationship between the TMB and the TS score (Additional file 5: Figure S5C and S5D). However, we found that TMB was a protective prognostic factor for BLCA patients and that combining the TS score with TMB improved the predictive value of TMB for OS time among BLCA patients (Additional file 5: Figure S5E and F). Finally, we found that compared to the low TS score group, the high TS score group had higher TP53 and lower KDM6A and FGFR3 mutation rates (Fig. 4H and I, Table 1).

**Table 1:** Association of the TS score with somatic variants

Gene symbol	High TS score (%)	Low TS score (%)	*p* value
TP53	113 (54%)	76 (39%)	0.0028
TTN	84 (40%)	76 (39%)	0.8393
KMT2D	59 (28%)	43 (22%)	0.1697
MUC16	48 (23%)	49 (25%)	0.6421
ARID1A	52 (25%)	45 (23%)	0.7270
KDM6A	36 (17%)	53 (27%)	0.0167
PIK3CA	42 (20%)	41 (21%)	0.9020
SYNE1	33 (16%)	37 (19%)	0.4313
RB1	38 (18%)	21 (11%)	0.0478
KMT2C	29 (14%)	29 (15%)	0.7787
HMCN1	33 (16%)	25 (13%)	0.4780
FGFR3	8 (4%)	47 (24%)	< 0.0001
RYR2	23 (11%)	33 (17%)	0.1124
MACF1	27 (13%)	27 (14%)	0.8838
EP300	31 (15%)	23 (12%)	0.3842
FLG	31 (15%)	21 (11%)	0.2376
FAT4	33 (16%)	18 (9%)	0.0520
STAG2	21 (10%)	27 (14%)	0.2820
ATM	25 (12%)	21 (11%)	0.7554
OBSCN	19 (9%)	27 (14%)	0.1586

### The TS score had significant prognostic value in BLCA

**Fig. 5:**
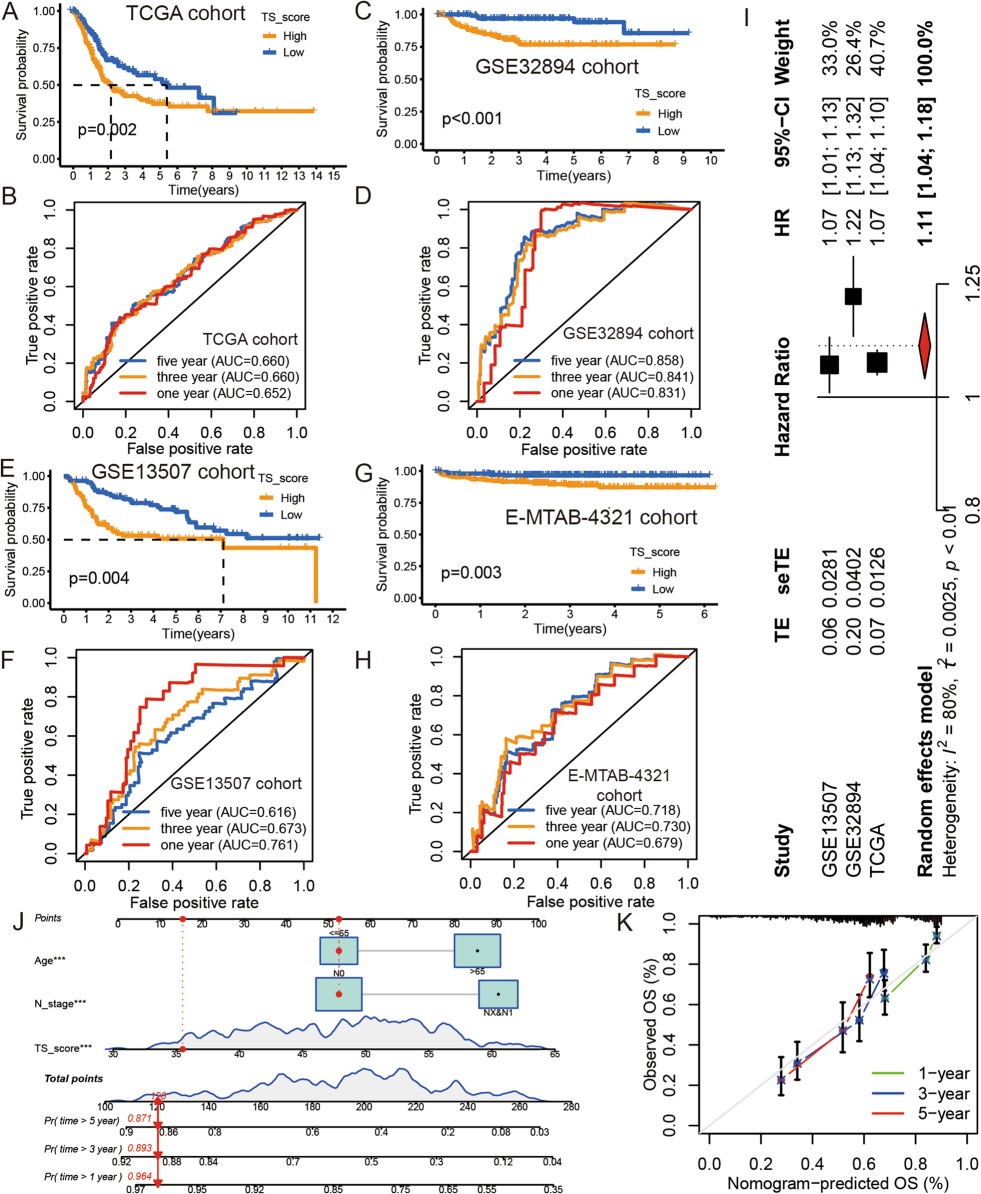
The prognostic value of TS score in BLCA. **A**, **C**, **E**, and **G** Kaplan–Meier curves for low and high TS score groups in TCGA BLCA cohorts (**A**). Log-rank test, *p* = 0.002; in GSE32894 cohort (**C**). Log-rank test, *p* < 0.001; in GSE13507 cohort (**E**). Log-rank test, *p* = 0.004; and in E-MTAB-4321 cohort (**G**). Log-rank test, *p* = 0.003. **B**, **D**, **F**, and **H** The ROC analysis of TS score in TCGA BLCA cohorts (**B**). AUC = 0.652, 0.660, and 0.660 at 1, 3, and 5 year; in GSE32894 cohort (**D**). AUC = 0.831, 0.841, and 0.851 at 1, 3, and 5 year; in GSE13507 cohort (**F**). AUC = 0.761, 0.673, and 0.616 at 1, 3, and 5 year; and in GSE13507 cohort (**H**). AUC = 0.718, 0.730, and 0.679 at 1, 3, and 5 year. **I** The meta-analysis of the TS score’s HR in three cohorts. **J** The nomogram based on TS score and clinical variates. **K** Calibration curve of the nomogram in TCGA BLCA cohorts

Having shown the correlation between the TS score, CSCs, and EMT status, we further explored the prognostic value of the TS score and discovered that the patients from the low TS group had longer OS times than patients from the high TS group in the TCGA BLCA cohort (Fig. 5A,  *p* = 0.002). We then confirmed this result in the GSE32894 (Fig. 5C, *p* < 0.001) and GSE13507 (Fig. 5E, *p* = 0.004) cohorts. We further confirmed the predictive value of disease-free survival (DFS) in the E-MTAB-4321 cohort (Fig. 5G, *p* = 0.003). The ROC curve also suggested that the TS score could predict the OS/DFS time of patients in the TCGA BLCA, GSE32894, GSE13507, and E-MTAB-4321 cohorts at 1, 3, and 5 years. (Fig. 5B, 5D, 5F, and 5H). To further identify the predictive value of the TS score in BLCA, we performed univariate Cox analysis and meta-analysis to calculate the hazard ratio (HR) in the three datasets (Fig. 5I). The results further indicate that the TS score was a reliable prognostic marker.

This finding was further confirmed by univariable and multivariable Cox analysis of the TS score and clinical factors, including age, sex, pathological stage, T stage, N stage, and M stage, in the TCGA BLCA cohorts (Additional file 20: Table S9). The results indicated that the TS score is an effective and reliable prognostic signature for BLCA patients. We also found that the TS score increased as T stage, M stage, and pathological stage increased. Moreover, we found that white people have higher TS scores than black and Asian people, and we think this is why the incidence is higher in white people than in black people [8]. However, the TS score did not differ significantly based on age, sex, or N stage (Additional file 6: Figure S6A–G). To help clinicians predict the prognosis of BLCA patients, we used the TS score and clinical variables (age and N stage) with an independent prognostic value plot in a nomogram based on the TCGA BLCA cohort (Fig. 5J). Finally, we further evaluated the ability of the nomogram to predict prognosis by calibration curves, ROC curves, and K–M survival curves. The analysis of the efficacy of the nomogram demonstrated that it could accurately predict the OS time of BLCA patients (Fig. 5K and Additional file 6: Figure S6H–K).

### The TS score could reflect CSC levels, TME characteristics, and patient prognoses in many types of cancers

**Fig. 6:**
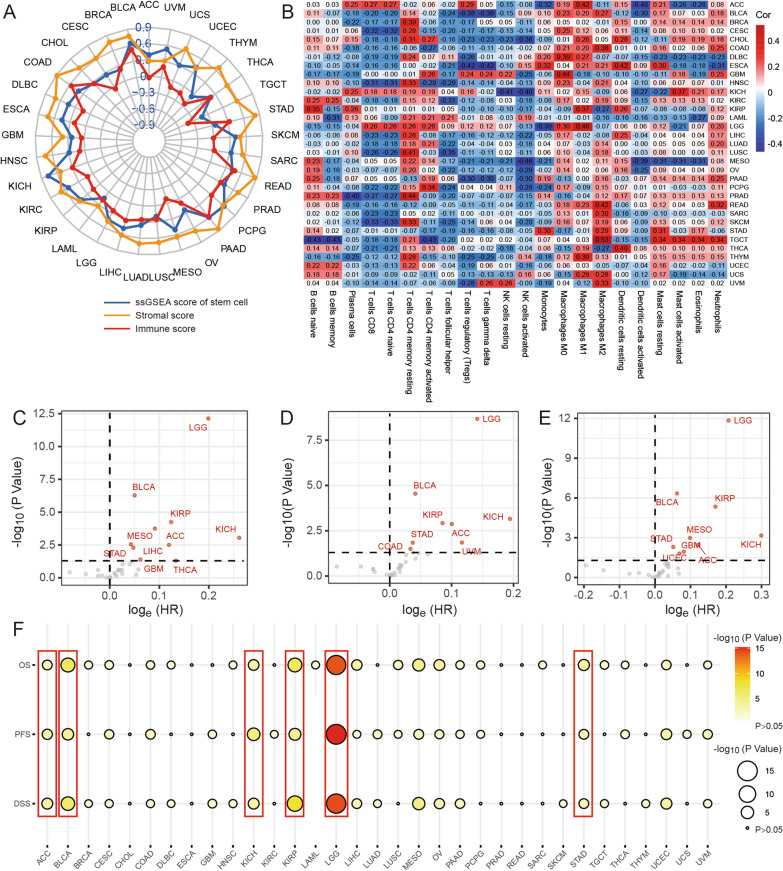
The TS score could estimate the TME, CSCs, immune infiltration, and prognosis in 33 types of cancer. **A** The radar map of correlation between TS score, ssGSEA score of stem cell gene set, stromal score, and immune score in 33 types of cancer. **B** The heatmap of correlation between TS score and 22 immune cells in 33 types of cancer. Red represents positive correlation, and blue represents negative correlation. **C** The volcano plot of univariate cox analysis between TS score and OS in 33 types of cancer. *p* < 0.05. **D** The volcano plot of univariate cox analysis between TS score and PFS in 32 types of cancer. *p* < 0.05. **E** The volcano plot of univariate Cox analysis between TS score and DSS in 32 types of cancer. *p* < 0.05. **F** The bubble diagram of P value from OS, PFS, DSS survival analysis between high and low TS score groups in each type of cancer. Log-rank test, *p* < 0.05. The cancer with red line means p value from univariate cox analysis of OS, PFS, and DSS, and log-rank test of OS, PFS, and DSS survival analysis less than 0.05

Previous studies have revealed that the TS process occurs in many cancer types. To further estimate the biological significance of the TS score in other tumor types, we calculated the TS score for all TCGA cohorts, including 33 types of cancer. Then, we further explored the correlations between CSCs, TME characteristics, immune infiltration, and prognoses. The results indicated that the TS score was closely related to the stem cell ssGSEA score, stromal score, and immune score in other cancer types (Fig. 6A). As the heatmap of immune cell content displayed, the relationship between the TS score and immune infiltration status was different in different tumors. However, we found that resting memory CD4 T cells and M2 macrophages were positively correlated with the TS score, while CD8 T cells and naive CD4 T cells were negatively correlated with the TS score in many cancer types (Fig. 6B).

Since the TS score has an outstanding predictive value for BLCA prognosis, we further estimated the prognostic value of the TS score in all TCGA cohorts by univariate Cox regression and K–M survival analyses. We found that the TS score was also associated with OS, progression-free survival (PFS), and disease-free survival (DSS) time in adrenocortical carcinoma (ACC), kidney chromophobe (KICH), kidney renal papillary cell carcinoma (KIRP), brain lower grade glioma (LGG), and stomach adenocarcinoma (STAD) in the TCGA cohort (Fig. 6C–F and Additional file 7: Figure S7, Additional file 8: Fig. S8, Additional file 9: Fig. S9, Additional file 21: Table S10). These results suggest that TS plays an import role in tumor progression.

### Predictive value of the TS score in immunotherapy

**Fig. 7:**
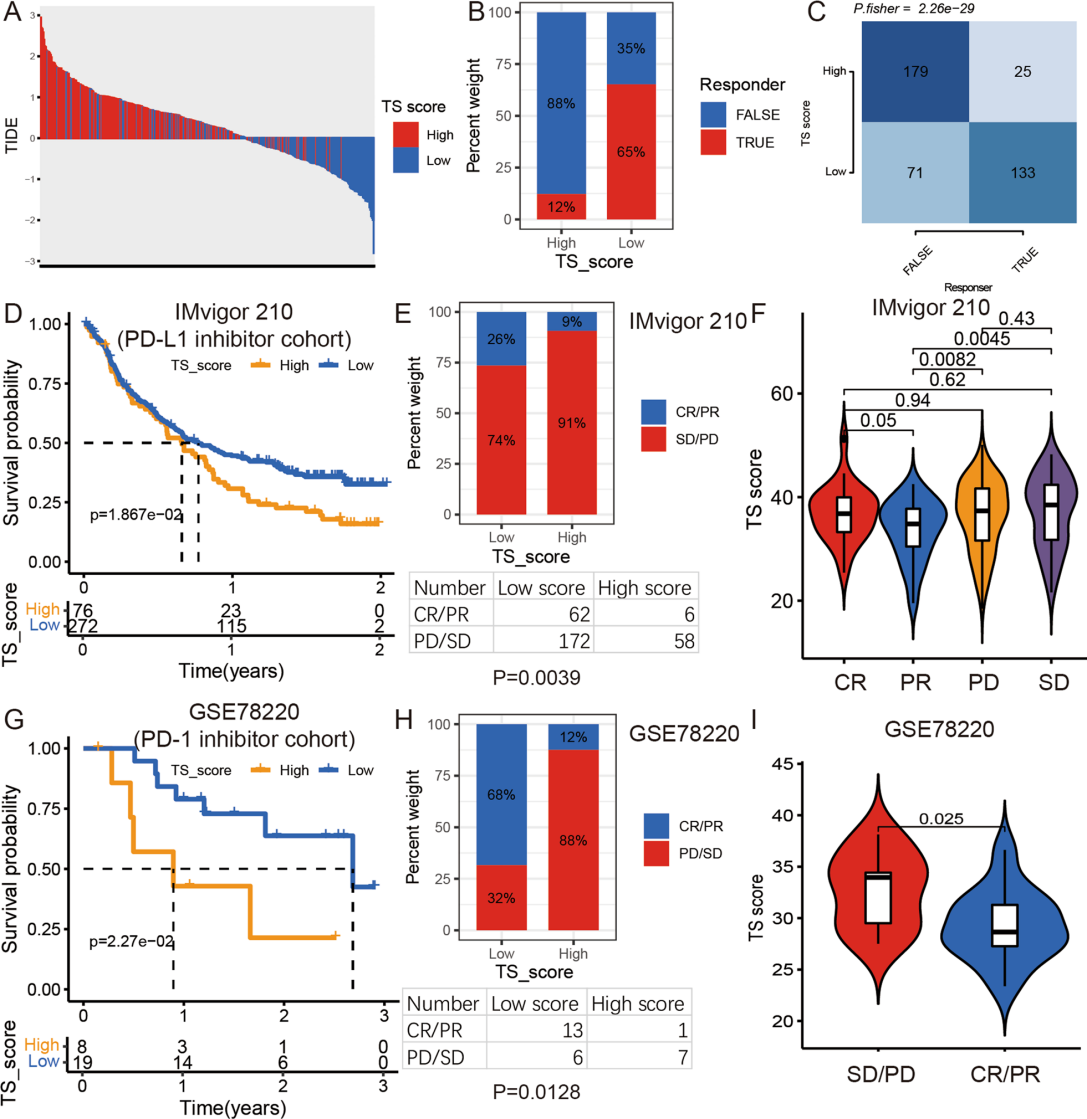
The relationship between TS score and immunotherapy response. **A** The TIDE score of each patient in high and low TS score groups. **B** Rate of response to immunotherapies predicted by TIDE in high or low TS score groups. **C** The count of patients in high or low TS score groups and response to immunotherapies true or false. Fisher’s exact test, *p* < 0.001. **D** Kaplan–Meier curves for low and high TS score groups in IMvigor 210 cohort. Log-rank test, *p* = 0.019. **E** Rate of clinical response (complete response [CR]/partial response [PR] and stable disease [SD]/progressive disease [PD]) to various immunotherapies in high or low TS score groups in the IMvigor 210 cohort. Fisher’s exact test, *p* = 0.0039. **F** Violin plot of TS score for PD, SD, PR, and CR groups in the IMvigor 210 cohort. **G** Kaplan–Meier curves for low and high TS score groups in the GSE78220 cohort. Log-rank test, *p* = 0.027. **H** Rate of clinical response (CR/PR and SD/PD) to various immunotherapies in high or low TS score groups in the GSE78220 cohort. Fisher’s exact test, *p* = 0.0128. **I** Violin plot of TS score for PD/SD and PR/CR groups in the GSE78220 cohort

In the above analysis, we found different immune infiltration levels in different TS statuses. Therefore, we further proposed that the TS status of tumors might be associated with the effect of immunotherapy in cancer. First, we predicted the response to immunotherapy of each tumor based on tumor immune dysfunction and exclusion (TIDE, http://tide.dfci.harvard.edu/) in the TCGA BLCA cohort. We found that patients with low TS scores had lower TIDE scores than those with high TS scores, meaning that low TS score patients were more efficient with immunotherapy (Fig. 7A–C). Then, we further calculated the TS score for BLCA patients in the immunotherapy cohort (Imvigor210) and found that BLCA patients from the low TS score group had a better prognosis and higher reaction rate to PD-L1 inhibitor treatment than patients from the high TS score group (Fig. 7D–F). We then downloaded melanoma sample data from the PD-1 inhibitor therapy cohort (GSE78220) and obtained the same result, with patients with high TS scores demonstrating a lower PD-1 inhibitory therapy response rate than those with low TS scores (Fig. 7G–I). All these results showed that the TS score could be an outstanding signature to predict the response to immunotherapy.

### The TS score could reflect the EMT and CSC characteristics of cell lines

**Fig. 8:**
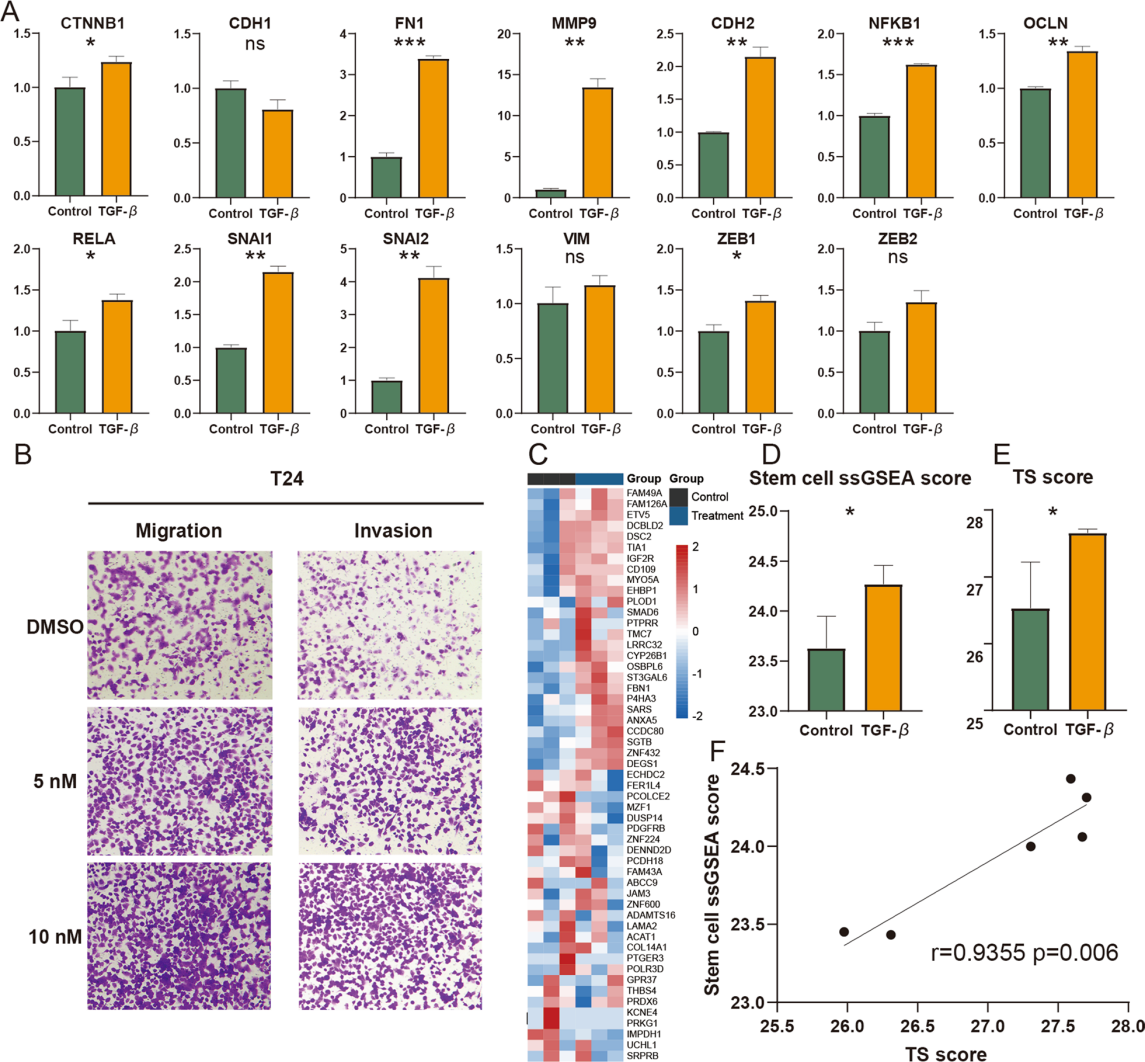
The TS score can reflect the EMT and stemness characteristics of T24. **A** The comparison of EMT biomarkers’ expression levels between T24 with and without TGF-β treatment. **B** The transwell assay of T24 with or without TGF-β treatment. **C** The heatmap of TS-related gene expression in T24 with or without TGF-β treatment. **D** The comparison of TS score between T24 with and without TGF-**β** treatment. **E** The comparison of stem cell ssGSEA score between T24 with and without TGF-β treatment. **F** The correlation between TS score and stem cell ssGSEA score in T24 cell line. Student’s t test, *p < 0.05, **p < 0.01, ***p < 0.001

TGF-β is a well-known EMT inducer and has been applied to investigate the EMT mechanism of cells [2, 38]. Moreover, TGF-β treatment significantly increases the stemness of cell lines [39–42]. To further directly confirm whether the TS score could predict the EMT and stemness characteristics of cells, we compared the TS score between cell with and without TGF-β treatment. First, the migration, invasion, and wound filling assays of T24 cells indicated that TGF-β could significantly increase the invasion ability of BLCA cells (Fig. 8B and Additional file 10: Figure S10A), and the cell viability assay suggested that TGF-β had little effect on the growth of T24 cells (Additional file 10: Figure S10B). Then, the RT‒qPCR trial verified that TGF-β could improve the expression of EMT marker genes and promote the EMT process of BLCA (Fig. 8A). Finally, we performed RNA-seq for T24 cells treated with TGF-β and calculated the TS score and ssGSEA score of the stem gene set. By displaying the expression level of TS-related genes and performing differential analysis and correlation analysis of the TS score and stem cell gene set ssGSEA score, we found that TGF-β could improve the stemness and TS score of BLCA and further validated that the TS score could reflect the EMT process and stemness of BLCA (Fig. 8C-8F).

To further validate whether the TS score could reflect the EMT status of tumors, we calculated the TS score for other cell lines treated with TGF-β in public datasets. Although GSE98979 only included the gene expression information of 35 TS-related genes, the TS scores of lung cancer cell lines were increased with TGF-β treatment, and this increase was time-dependent (Additional file 11: Figure S11A and B). This variation in TS scores was in accordance with the changes in EMT characteristics in the original study. Moreover, we found that the TS score increased not only in the breast cancer cell line (Additional file 11: Figure S11C) but also in the normal breast tissue cell line (Additional file 11: Figure S11D). Finally, we analyzed datasets of pancreatic cancer cell lines treated with TGF-β and found that the TS score of cell line increased with TGF-β treatment (Additional file 11: Figure S11E). These results were in agreement with those of the pan-cancer analysis of the prognostic value of the TS score and indicated that our TS score could accurately predict the EMT and stemness characteristics of cells and had application value in cell biology research.

## Discussion

In most malignancies, the subsequent outgrowth of micrometastatic deposits into macroscopic metastases has the greatest impact on clinical progression, and EMT and tumor CSCs are the crucial biological characteristics associated with this progress. During the early stages of metastasis, tumor cells lose mediators of epithelial adhesion, such as E-cadherin, and exhibit a spindle cell morphology, as well as an increase in N-cadherin and vimentin [43, 44]. Stromal cells play an important role in accelerating EMT progression [45]. For example, tumor-associated fibroblasts (TAFs) can secrete TGF-β, IL-6, VEGF, and HGF to promote EMT [46–49]; tumor-associated macrophages (TAMs) can secrete cytokines and chemokines for matrix remodeling and immunosuppression and to promote angiogenesis, thereby promoting EMT [50, 51]; and T cells can decrease E-cadherin to accelerate EMT in tumors. [52, 53]. Then, in the late steps of metastasis, the colonization of tumor cells is likely to require adaptation of disseminated cancer cells to the microenvironment of foreign tissue. Therefore, tumor cells undergoing EMT require CSCs with self-renewal and redifferentiation capabilities. In this process, many signaling pathways, such as the Wnt and Notch pathways, as well as the TME, play vital roles [54, 55]. EMT and CSC translation are not independent. The mesenchymal cells from EMT are the main source of CSC translation and require a similar stromal environment [56, 57]. Ultimately, the process of mesenchymal–epithelial transition (MET) leads to distant transfer of tumor cells. EMT, CSCs, and MET are closely related to each other and associated with patient prognosis [58–60]. In NMIBC, cancer cells are confined to the epithelium of the bladder and have weak invasive abilities. In this phase, the speed of tumor development is slow and mild. Through EMT, some epithelial tumor cells transform into CSCs with strong proliferative and invasive abilities, and patients ultimately develop MIBC [4].

In our study, we found that EMT progression was associated with the CSCs of BLCA via single-cell bioinformatic analysis. We first explored the cell subtypes and differentiation trajectories of bladder tumors.Although the level of tissue stem cells in BLCA was low, CSCs from EMT was a significant prognostic factor. Then, TS biomarkers were extracted through dimension reduction analysis, differential analysis, and analysis of the cell development trajectory based on BLCA single-cell mRNA sequencing data. The prognostic analysis revealed that the EMT process and CSC content could decrease the OS time of BLCA patients. The single-cell sequencing data from CD45-BLCA cells did not reveal immune status. Thus, we performed clustering analysis and obtained three TS clusters and two TS-related gene clusters. TS cluster A corresponded with TS-related gene cluster A and had lower EMT progression and CSC content and better prognoses, while TS cluster B and TS cluster C corresponded with TS-related gene cluster B and had more active EMT progression, higher CSC content, and worse outcomes. We also found that the expression levels of TS biomarkers were consistent among BLCA samples. After the clustering analysis, we further extracted the TS characteristics of BLCA and obtained 61 TS-related genes. Finally, we constructed a TS score to estimate tumor EMT progression and CSCs. The correlation of the TS score with EMT biomarkers and the ssGSEA score of stem cells further confirmed that the TS score could represent EMT progression and CSCs in BLCA.

Then, we found that the group with a high TS score had a worse prognosis than the group with a low TS score through prognostic analysis. According to previous studies, these results further confirmed that TS was a vital factor driving tumor growth and metastasis [4, 12]. These results also indicated that the TS score had outstanding predictive value for BLCA prognosis. Moreover, we calculated the TS score for 33 cancer types and found that the TS score was a risk factor in most cancers, particularly ACC, BLCA, KICH, KIRP, LGG, and STAD. These results further proved that the TS score was an efficient prognostic biomarker across cancers and that tumor cell invasiveness and metastasis were associated with TS in ACC, BLCA, KICH, KIRP, LGG, and STAD [61–63].

The TME includes stromal cells and immune cells and plays a crucial role in tumor development. Some studies have indicated that the TME can impact EMT and influence the characteristics of CSCs [2]. For example, integrins from the extracellular matrix can impact EMT in tumors [64]. We further explored the relationship between stromal cells and TS and found that the TS score was positively correlated with the stromal score in many cancers. Therefore, some molecular compounds and cellular components in the extracellular matrix likely promote TS, increasing the proliferation and invasion capabilities of tumor cells [65, 66]. Moreover, we found multiple relationships between immune cells and TS in different cancer types. CD8+ T cells and CD4+ naive T cells were negatively correlated with TS, while M0 and M2 macrophages were positively correlated with TS in many cancer types. Thus, macrophages might promote TS in some cancers [67, 68]. However, the deeper mechanics of this phenomenon needed further investigation.

Many gene multiomics studies suggest that DNA mutations play a crucial role in EMT in tumors. For example, induction of ZEB allows the expansion of an EMT-competent unique cellular subpopulation in esophageal cancer [69]. Here, we found that OS time increased as the TMB increased in BLCA. A high TMB and high tumor heterogeneity likely stimulate immune cells and increase their ability to kill tumor cells. While we did not find a significant relationship between TS and DNA mutation burden, we found that TP53 mutation rates were higher, while ARID1A and FGFR3 were lower in the high TS score group than in the low TS score group in the TCGA BLCA cohort. Therefore, TP53 mutation likely promotes TS in tumor cells and impacts BLCA prognosis; however, these hypotheses needed to be confirmed with further in vitro experiments [70].

Immunotherapy is an effective treatment for BLCA. For high-risk NMIBC, bladder infusion therapy with BCG is still the most efficient treatment to inhibit the recurrence of tumors after transurethral resection [71]. For metastatic BLCA, chemotherapy and immunotherapy are the main treatments. Immunotherapy has fewer side effects than chemotherapy and is less likely to result in BLCA tolerance to treatment [17]. Here, we found that TS was associated with immune cell levels and the expression of checkpoint genes. Therefore, we further explored the relationship between TS and immunotherapy. We found that tumors with high TS scores were less sensitive to PD-1/L1 inhibitor treatment. On the one hand, a high TS score likely increased proliferation and invasion capabilities. On the other hand, a high TS score meaned lower tumor CD8+ T cell content and higher tumor macrophage content [72, 73]. Thus, these tumors with high TS scores were not sensitive to immunotherapy.

Finally, because TGF-β can promote the EMT and stemness characteristics of cells, we assessed cell lines treated with TGF-β to further verify our hypothesis that the TS score can reflect the EMT and CSC features of multiple types of cells. We found that the TS score can accurately reflect the changes in cell physiological characteristics with TGF-β treatment, although these cells were from different parts of the body and from different types of tissue (tumor or normal tissue). This means that the TS score is a valuable index in EMT and stem cell studies.

Compared with other studies, we first explored the biological features of TS and TS-related gene clusters in BLCA based on single-cell and bulk mRNA sequencing data and microarray data from multiple cohorts. Then, we further identified TS-related genes and constructed a TS scoring system to estimate and quantify TS. In addition to the prognostic value of the TS score, we also found that the TS score was associated with the response to immunotherapy. Finally, we verified that the TS score is an efficient index for EMT and CSC research by RT‒qPCR, mRNA sequencing of cell lines, and transwell assays. However, this study has limitations. First, some of the conclusions need to be further confirmed with in vitro and in vivo experiments. Second, the TS score cannot directly compare different cohorts without batch correction because of the batch effect.

## Conclusions

In brief, we identified three disparate TS clusters and two TS-related gene clusters using TS biomarkers identified from a single-cell analysis and established a TS score for BLCA. The TS score is associated with tumor EMT, CSCs, tumor stromal cells, tumor immune infiltration, and TP53 mutation characteristics and is an efficient index for estimating TS and predicting prognosis and therapeutic responsiveness. Thus, the TS score may aid research into EMT and CSCs and facilitate the future development of personalized immunotherapy approaches for many cancer types.

## Supplementary Information

**Additional file 1: Figure 1**. Overview of single-cell and prognostic value of each type cell’s biomarker in BLCA tissue. (A) The workflow of this study. (B) The t-SNE plot of all the single cells, with each color coded for 7 major cell clusters. (C) Differentiation trajectory of 7 cell clusters in BLCA. The violin plot of gene expression of RGS5 (D), KRT7 (E), and CD44 (F) in cluster0-6. (G) The bubble plot of gene expression of RGS5, KRT7, and CD44 in cluster 0-6. The RGS5 (H), KRT7 (I), and CD44 (J) expression of each cell in the t-SNE map.

**Additional file 2: Figure 2**. (A) Kaplan–Meier curves for high/low state 2 ssGSEA score groups in TCGA BLCA cohort. Log-rank test, p = 0.068. (B) Kaplan–Meier curves for high/low state 3 ssGSEA score groups in TCGA BLCA cohort. Log-rank test, p = 0.734. (C) Kaplan–Meier curves for high/low chondrocytes ssGSEA score groups in TCGA BLCA cohort. log-rank test, p = 0.048. The GO (D: BP [Biological Process]; E: MF [Molecular Function]; F: CC [Cellular Component]) and KEGG (G) function enrichment analysis of TS biomarkers.

**Additional file 3: Figure 3**. Consensus clustering of BLCA distinct TS clusters. (A) CDF curve. (B) CDF Delta area curve. Delta area curve of consensus clustering, indicating the relative change in area under the cumulative distribution function (CDF) curve for each category number k compared with k-1. The horizontal axis represents the category number k and the vertical axis represents the relative change in area under CDF curve. (C-G) Consensus matrixes of BLCA samples for each k (k = 2–6) from TCGA BLCA cohort based on expression abundance of TS biomarkers, displaying the clustering stability using 1000 iterations of clustering. (H) Principal Component Analysis (PCA) of TCGA BLCA sample based on expression abundance of TS biomarkers.

**Additional file 4: Figure 4**. Consensus clustering of BLCA distinct TS-related gene clusters. (A) Venn diagram of DEGs between three TS clusters. (B) CDF curve. (C) CDF Delta area curve. Delta area curve of consensus clustering, indicating the relative change in area under the cumulative distribution function (CDF) curve for each category number k compared with k-1. The horizontal axis represents the category number k and the vertical axis represents the relative change in area under CDF curve. (D-H) Consensus matrixes of BLCA samples for each k (k = 2–6) from meta cohort based on expression abundance of TS-related genes, displaying the clustering stability using 1000 iterations of clustering. (I) Uniform manifold approximation and projection (UMAP) of TCGA BLCA sample based on expression abundance of TS-related genes.

**Additional file 5: Figure 5**. (A) Boxplot of expression of 14 checkpoints gene for low and high TS score groups in TCGA BLCA cohort. (B) Boxplot of 22 immune cells for low and high TS score groups in TCGA BLCA cohort. Wilcox test, *p < 0.05, **p < 0.01, ***p < 0.001. (C) TMB difference in the high and low TS score groups in TCGA BLCA cohort. Wilcoxon test, p = 0.36. (D) The correlation between TMB and TS score. Spearman, Cor = - 0.08, p = 0.11. (E) Kaplan–Meier curves for high and low TMB groups in TCGA BLCA cohort. Log-rank test, p = 0.010. (F) Kaplan–Meier curves for patients stratified by both TMB and TS score in TCGA BLCA cohort. Log-rank test, p<0.001.

**Additional file 6: Figure 6.** The differential analysis of age (A), sex (B), pathological stage (C), T stage (D), M stage (E), N stage (F), race (G) between high and low TS score groups in TCGA BLCA cohort. Wilcox test: p (age) = 0.052, p (sex) = 0.059, p (pathological stage) < 0.001, p (T stage) < 0.001, p (M stage) < 0.001, p (N stage) = 0.061 and p (race) < 0.001. The ROC analysis of nomogram in TCGA BLCA cohort. AUC = 0.743 (H), 0.750 (I), and 0.774 (J) at 1, 3, and 5 year. (K) Kaplan–Meier curves for high and low nomogram points in TCGA BLCA cohort. Log-rank test, p < 0.001.

**Additional file 7: Figure 7**. Kaplan–Meier curves of OS for high and low TS score patients in 23 types of cancer. Log-rank test, p < 0.05.

**Additional file 8: Figure 8**. Kaplan–Meier curves of PFS for high and low TS score patients in 22 types of cancer. Log-rank test, p < 0.05.

**Additional file 9: Figure 9**. Kaplan–Meier curves of DSS for high and low TS score patients in 21 types of cancer. Log-rank test, p < 0.05.

**Additional file 10: Figure 10**. The wound filling assay (A), and cell viability (CCK-8) assay of T24 with or without TGF-β treatment (B). ANOVA analysis, p > 0.05.

**Additional file 11: Figure 11**. The variation of TS score of cell lines after TGF-β treatment. (A) The comparison of TS score of H1975 with or without TGF-β treatment after 1 and 2 days. Student’s t test, *p < 0.05, **p < 0.01, ***p < 0.001. (B) The comparison of TS score of A549 after TGF-β 0, 4, 8, 16, 24, and 72 hours. ANOVA analysis, p = 0.003924. (C) The comparison of TS score of MCF7 with or without TGF-β treatment. Student’s t test, p = 0.02851 (D) The comparison of TS score of MCF10A with or without TGF-β treatment. Student’s t test, p < 0.00001 (E) The comparison of TS score of Panc-1 with or without TGF-β treatment. Student’s t test, p < 0.001030.

**Additional file 12: Table S1**. Clinical information of patients from TCGA BLCA cohort.

**Additional file 13: Table S2**. The primer sequence used in our study.

**Additional file 14: Table S3**. Biomarkers of different cell types, clusters, and states.

**Additional file 15: Table S4**. GO and KEGG gene function enrichment analysis of TS biomarkers.

**Additional file 16: Table S5**. The DEGs between three clusters.

**Additional file 17: Table S6**. The univariate Cox analysis of TS-related genes.

**Additional file 18: Table S7**. PCA analysis and PCA coefficient of TS-related genes to calculate TS score.

**Additional file 19: Table S8**. The GSEA analysis between low and high TS score group in TCGA BLCA cohort.

**Additional file 20: Table S9.** The univariate and multivariate Cox analysis of TS score and clinical variates.

**Additional file 21: Table S10**. The univariate and K–M survival analysis of TS score in 33 types of cancer.

## Data Availability

The public datasets used and/or analyzed during the current study are available from The Cancer Genome Atlas database (TCGA BLCA cohort: https://portal.gdc.cancer.gov), Gene Expression Omnibus (GSE146137: https://www.ncbi.nlm.nih.gov/geo/query/acc.cgi?acc=GSE146137, GSE78220: https://www.ncbi.nlm.nih.gov/geo/query/acc.cgi?acc=GSE78220, GSE13507: https://www.ncbi.nlm.nih.gov/geo/query/acc.cgi?acc=GSE13507, GSE98979 https://www.ncbi.nlm.nih.gov/geo/query/acc.cgi?acc=GSE98979, GSE17708 https://www.ncbi.nlm.nih.gov/geo/query/acc.cgi?acc=GSE17708, GSE101809 https://www.ncbi.nlm.nih.gov/geo/query/acc.cgi?acc=GSE101809, GSE124843 https://www.ncbi.nlm.nih.gov/geo/query/acc.cgi?acc=GSE124843, GSE23952 https://www.ncbi.nlm.nih.gov/geo/query/acc.cgi?acc=GSE23952, and GSE32894: https://www.ncbi.nlm.nih.gov/geo/query/acc.cgi?acc=GSE32894), ArrayExpress (E-MTAB-4321: https://www.ebi.ac.uk/biostudies/arrayexpress/studies/E-MTAB-4321?query=E-MTAB-4321), and University of California Santa Cruz (UCSC) Xena browser (https://xenabrowser.net) database (GDC Pan-Cancer cohort: https://gdc-hub.s3.us-east-1.amazonaws.com/download/GDC-PANCAN.htseq_fpkm-uq.tsv.gz). Finally, the RNA-seq of T24 cell line generated in our study is stored in Gene Expression Omnibus (GSE215947: https://www.ncbi.nlm.nih.gov/geo/query/acc.cgi?acc=GSE215947).

## Abbreviations

ACC: Adrenocortical carcinoma
AUC: Area under the ROC curve
BLCA: Bladder urothelial carcinoma
BRCA: Breast invasive carcinoma
CCK-8: Cell Counting Kit-8
CESC: Cervical squamous cell carcinoma and endocervical adenocarcinoma
CHOL: Cholangiocarcinoma
COAD: Colon adenocarcinoma
CR: Complete response
CSCs: Cancer stem cells
DEGs: Differentially expressed genes
DFS: Disease-free survival
DLBC: Lymphoid neoplasm diffuse large B cell lymphoma
EMT: Epithelial-to-mesenchymal transition
ESCA: Esophageal carcinoma
FPKM: Fragments per kilobase transcript per million mapped reads
GBM: Glioblastoma multiforme
GDSC: Genomics of drug sensitivity
GO: Gene Ontology
GSVA: Gene set variation analysis
HNSC: Head and neck squamous cell carcinoma
HR: Hazard ratio
KEGG: Kyoto Encyclopedia of Genes and Genomes
KICH: Kidney chromophobe
KIRC: Kidney renal clear cell carcinoma
KIRP: Kidney renal papillary cell carcinoma
K–M: Kaplan–Meier
LAML: Acute myeloid leukemia
LGG: Brain lower grade glioma
LIHC: Liver hepatocellular carcinoma
LUAD: Lung adenocarcinoma
LUSC: Lung squamous cell carcinoma
MESO: Mesothelioma
MET: Mesenchymal–epithelial transition
MIBC: Muscle-invasive bladder cancer
NMIBC: No muscle-invasive bladder cancer
OS: Overall survival
OV: Ovarian serous cystadenocarcinoma
PAAD: Pancreatic adenocarcinoma
PCA: Principal component analysis
PCPG: Pheochromocytoma and paraganglioma
PD: Progressive disease
PFS: Progression-free survival
PR: Partial response
PRAD: Prostate adenocarcinoma
RT-qPCR: Real-time quantitative polymerase chain reaction
READ: Rectum adenocarcinoma
ROC: Receiver operating characteristic
SARC: Sarcoma
SD: Stable disease
SKCM: Skin cutaneous melanoma
ssGSEA: Single-sample gene set enrichment analysis
STAD: Stomach adenocarcinoma
TCGA: The Cancer Genome Atlas
TGCT: Testicular germ cell tumors
TGF-β: Transforming growth factor-β
THCA: Thyroid carcinoma
THYM: Thymoma
TIDE: Tumor immune dysfunction and exclusion
TME: Tumor microenvironment
TPM: Transcripts per kilobase million
TS: Tumor stemness
t-SNE: T-distributed stochastic neighbor embedding
UCEC: Uterine corpus endometrial carcinoma
UCS: Uterine carcinosarcoma
UCSC: University of California Santa Cruz
UVM: Uveal melanoma
