# Supplementary figures and images for: Tumor stemness score to estimate epithelial-to-mesenchymal transition (EMT) and cancer stem cells (CSCs) characterization and to predict the prognosis and immunotherapy response in bladder urothelial carcinoma
Source: Stem Cell Res Ther. 2023 Feb 1;14:15. doi: 10.1186/s13287-023-03239-1 (PMC9890713; doi:10.1186/s13287-023-03239-1)

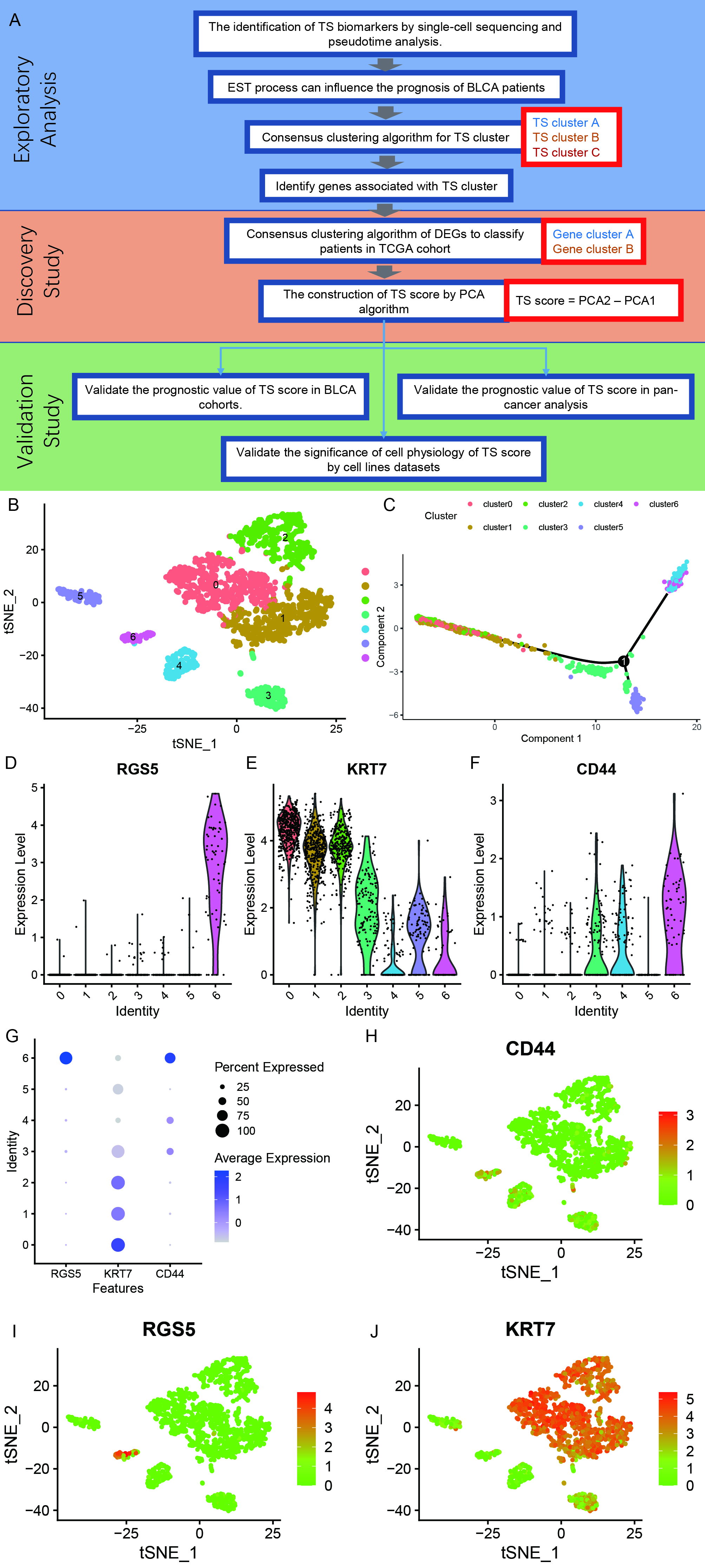

Supplement: Supplementary file 1 — Additional file 1: Figure 1. Overview of single-cell and prognostic value of each type cell’s biomarker in BLCA tissue. (A) The workflow of this study. (B) The t-SNE plot of all the single cells, with each color coded for 7 major cell clusters. (C) Differentiation trajectory of 7 cell clusters in BLCA. The violin plot of gene expression of RGS5 (D), KRT7 (E), and CD44 (F) in cluster0-6. (G) The bubble plot of gene expression of RGS5, KRT7, and CD44 in cluster 0-6. The RGS5 (H), KRT7 (I), and CD44 (J) expression of each cell in the t-SNE map. [file 13287_2023_3239_MOESM1_ESM.tif]

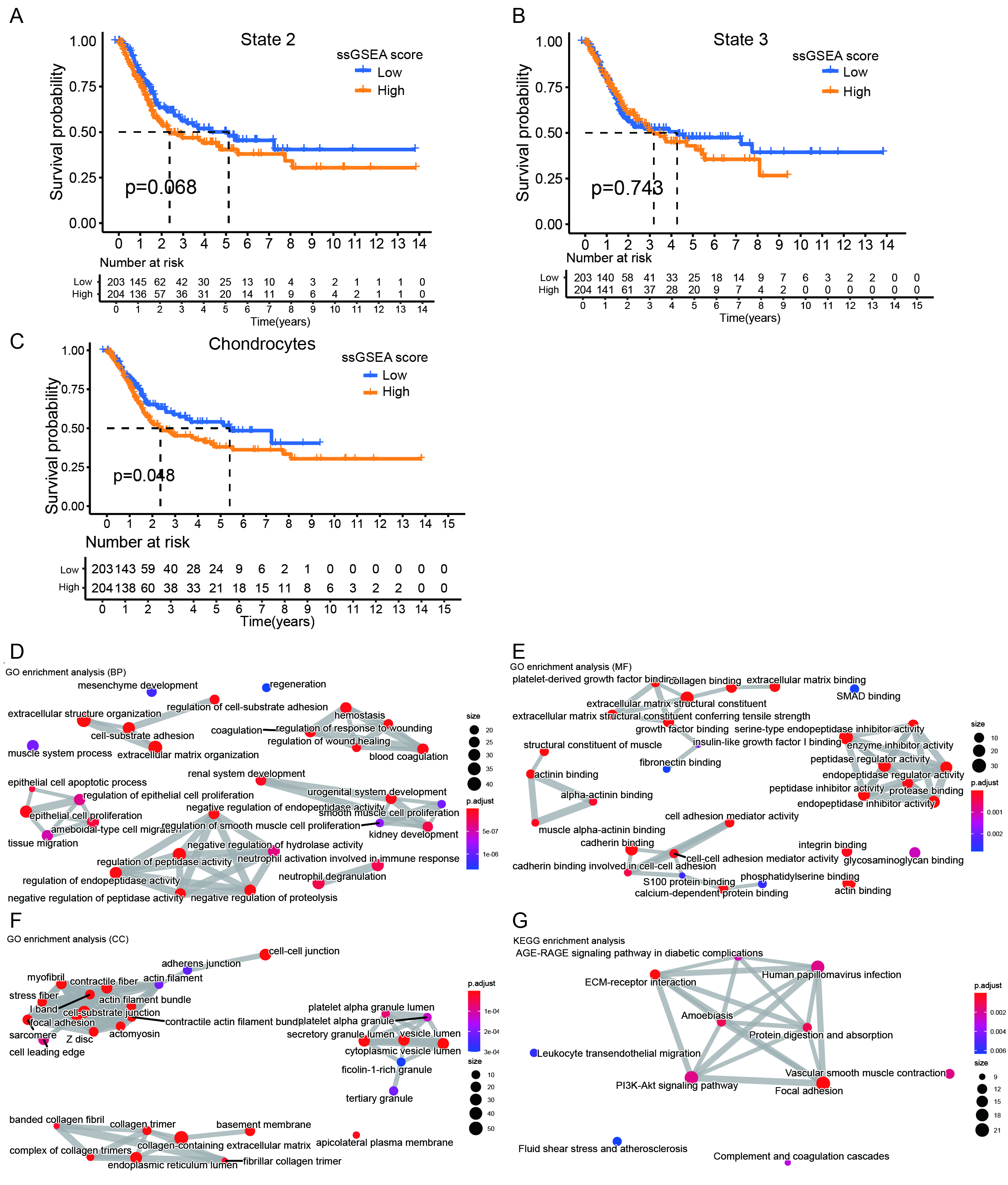

Supplement: Supplementary file 2 — Additional file 2: Figure 2. (A) Kaplan–Meier curves for high/low state 2 ssGSEA score groups in TCGA BLCA cohort. Log-rank test, p = 0.068. (B) Kaplan–Meier curves for high/low state 3 ssGSEA score groups in TCGA BLCA cohort. Log-rank test, p = 0.734. (C) Kaplan–Meier curves for high/low chondrocytes ssGSEA score groups in TCGA BLCA cohort. log-rank test, p = 0.048. The GO (D: BP [Biological Process]; E: MF [Molecular Function]; F: CC [Cellular Component]) and KEGG (G) function enrichment analysis of TS biomarkers. [file 13287_2023_3239_MOESM2_ESM.tif]

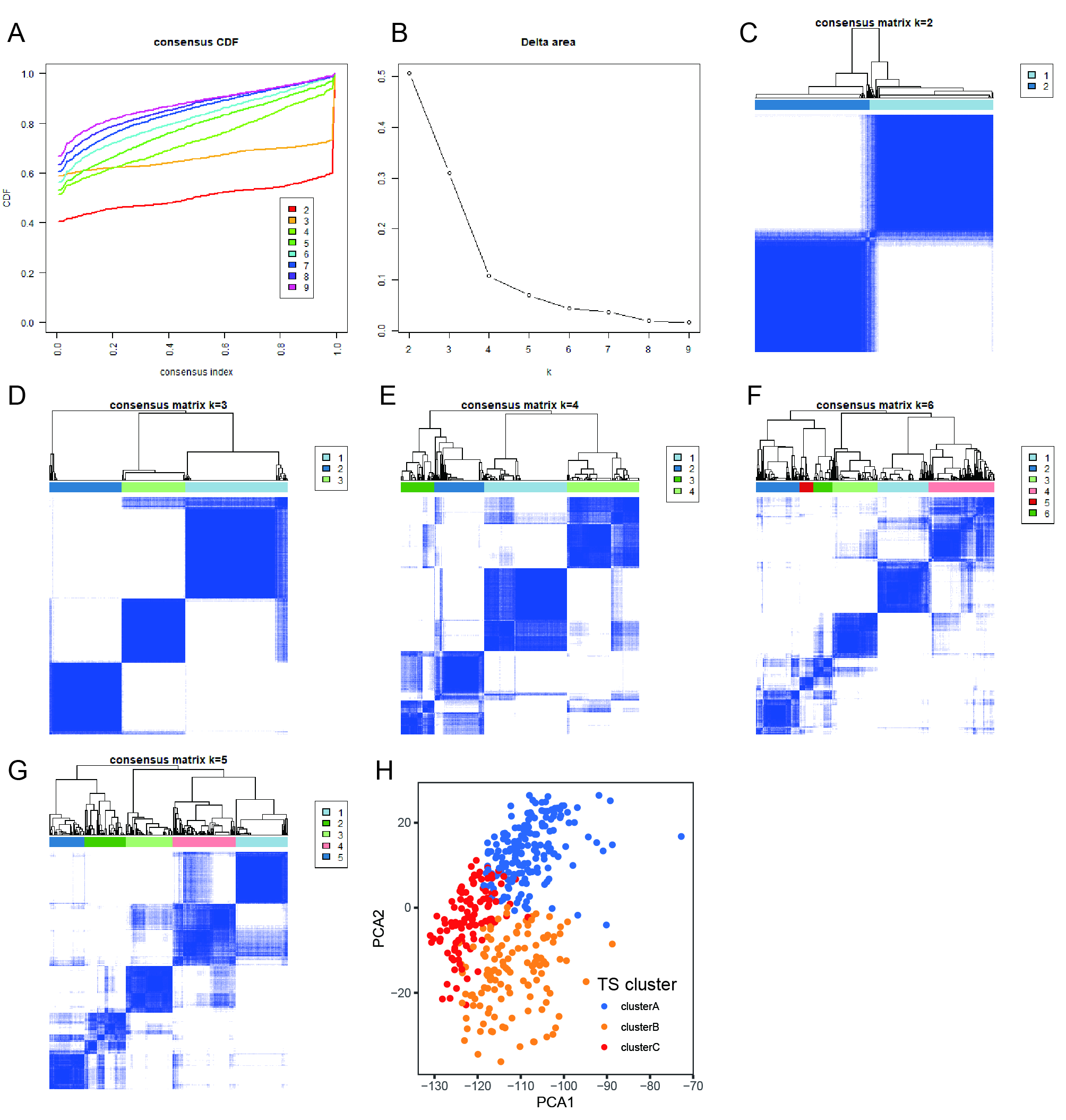

Supplement: Supplementary file 3 — Additional file 3: Figure 3. Consensus clustering of BLCA distinct TS clusters. (A) CDF curve. (B) CDF Delta area curve. Delta area curve of consensus clustering, indicating the relative change in area under the cumulative distribution function (CDF) curve for each category number k compared with k-1. The horizontal axis represents the category number k and the vertical axis represents the relative change in area under CDF curve. (C-G) Consensus matrixes of BLCA samples for each k (k = 2–6) from TCGA BLCA cohort based on expression abundance of TS biomarkers, displaying the clustering stability using 1000 iterations of clustering. (H) Principal Component Analysis (PCA) of TCGA BLCA sample based on expression abundance of TS biomarkers. [file 13287_2023_3239_MOESM3_ESM.tif]

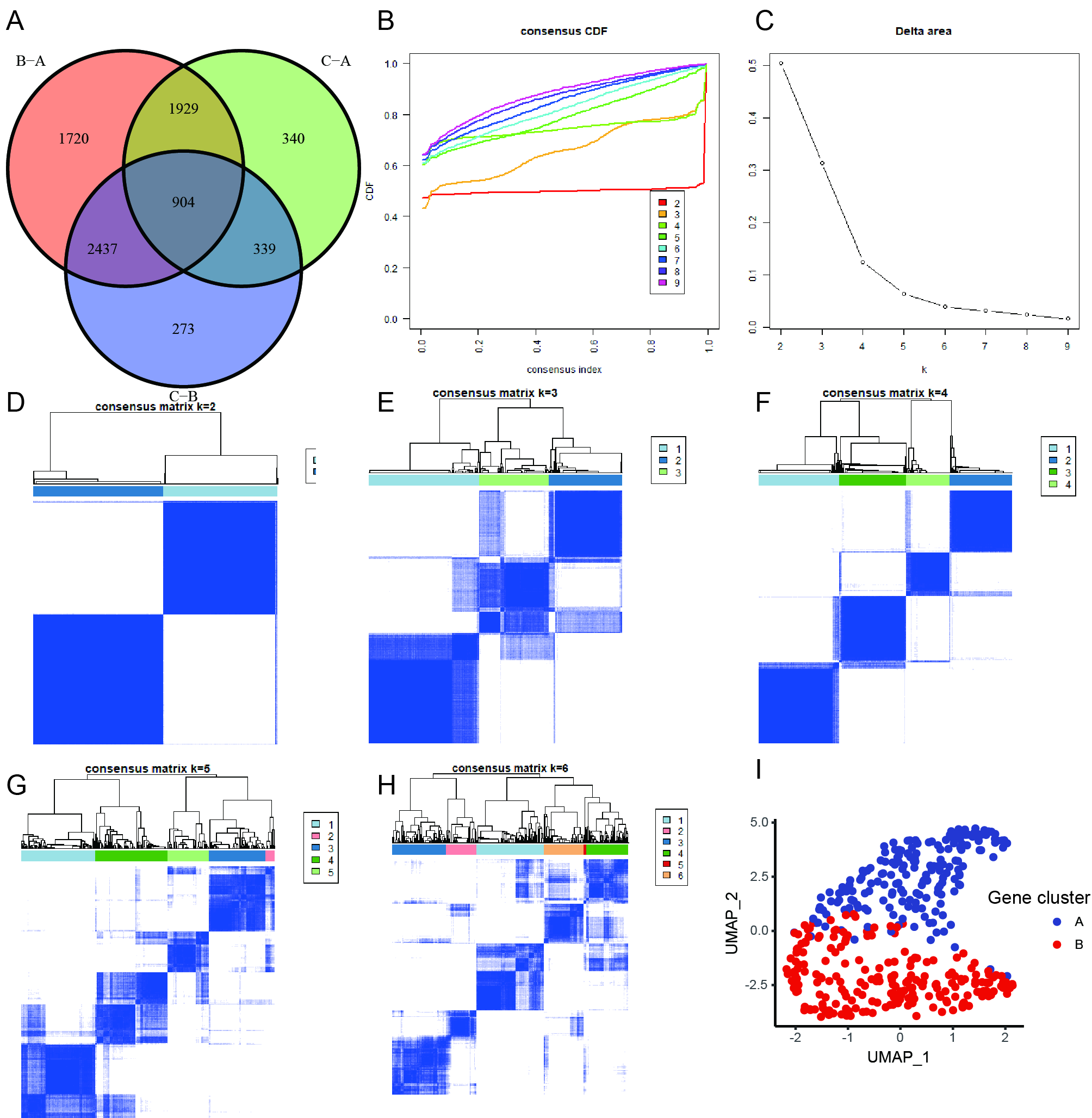

Supplement: Supplementary file 4 — Additional file 4: Figure 4. Consensus clustering of BLCA distinct TS-related gene clusters. (A) Venn diagram of DEGs between three TS clusters. (B) CDF curve. (C) CDF Delta area curve. Delta area curve of consensus clustering, indicating the relative change in area under the cumulative distribution function (CDF) curve for each category number k compared with k-1. The horizontal axis represents the category number k and the vertical axis represents the relative change in area under CDF curve. (D-H) Consensus matrixes of BLCA samples for each k (k = 2–6) from meta cohort based on expression abundance of TS-related genes, displaying the clustering stability using 1000 iterations of clustering. (I) Uniform manifold approximation and projection (UMAP) of TCGA BLCA sample based on expression abundance of TS-related genes. [file 13287_2023_3239_MOESM4_ESM.tif]

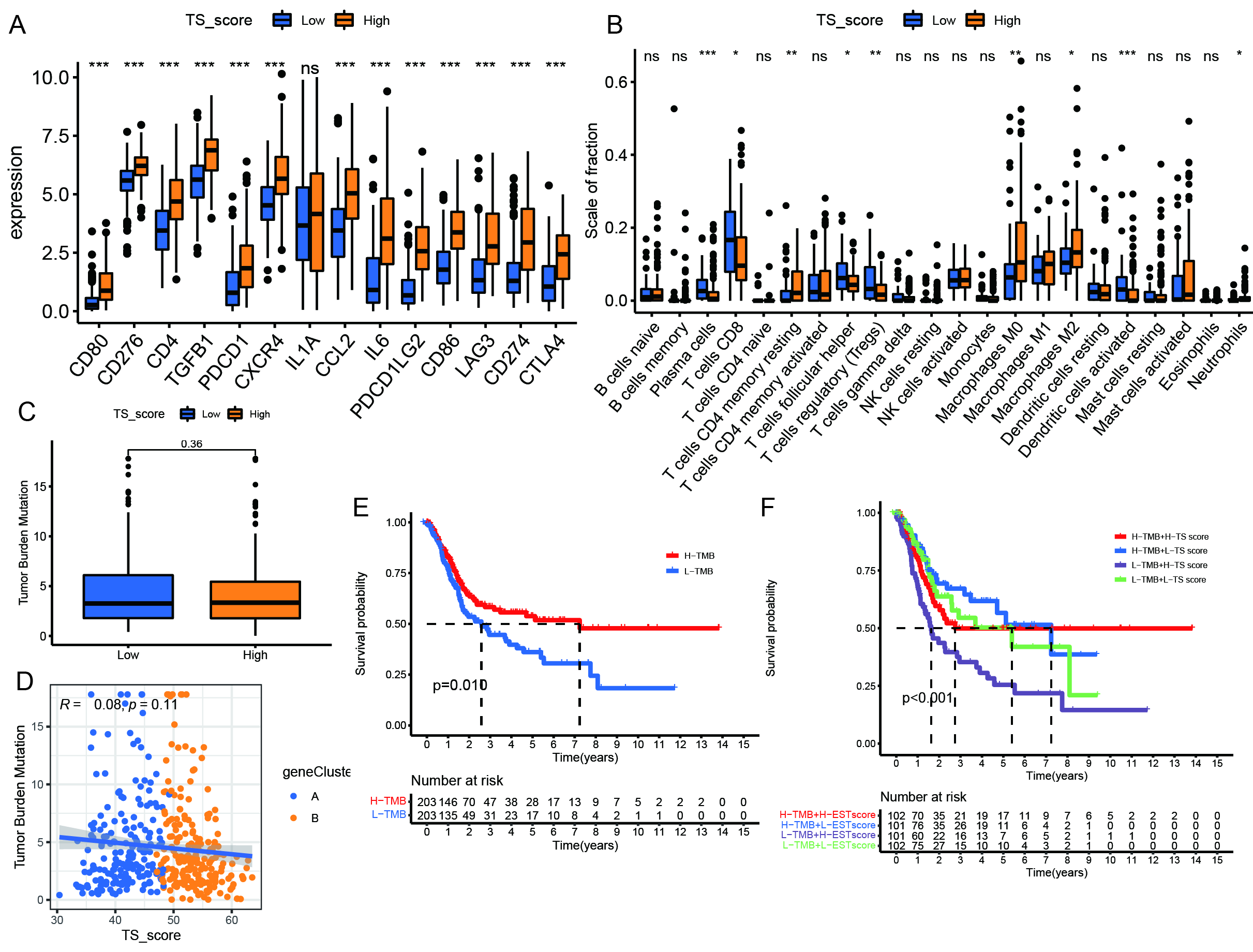

Supplement: Supplementary file 5 — Additional file 5: Figure 5. (A) Boxplot of expression of 14 checkpoints gene for low and high TS score groups in TCGA BLCA cohort. (B) Boxplot of 22 immune cells for low and high TS score groups in TCGA BLCA cohort. Wilcox test, *p < 0.05, **p < 0.01, ***p < 0.001. (C) TMB difference in the high and low TS score groups in TCGA BLCA cohort. Wilcoxon test, p = 0.36. (D) The correlation between TMB and TS score. Spearman, Cor = - 0.08, p = 0.11. (E) Kaplan–Meier curves for high and low TMB groups in TCGA BLCA cohort. Log-rank test, p = 0.010. (F) Kaplan–Meier curves for patients stratified by both TMB and TS score in TCGA BLCA cohort. Log-rank test, p<0.001. [file 13287_2023_3239_MOESM5_ESM.tif]

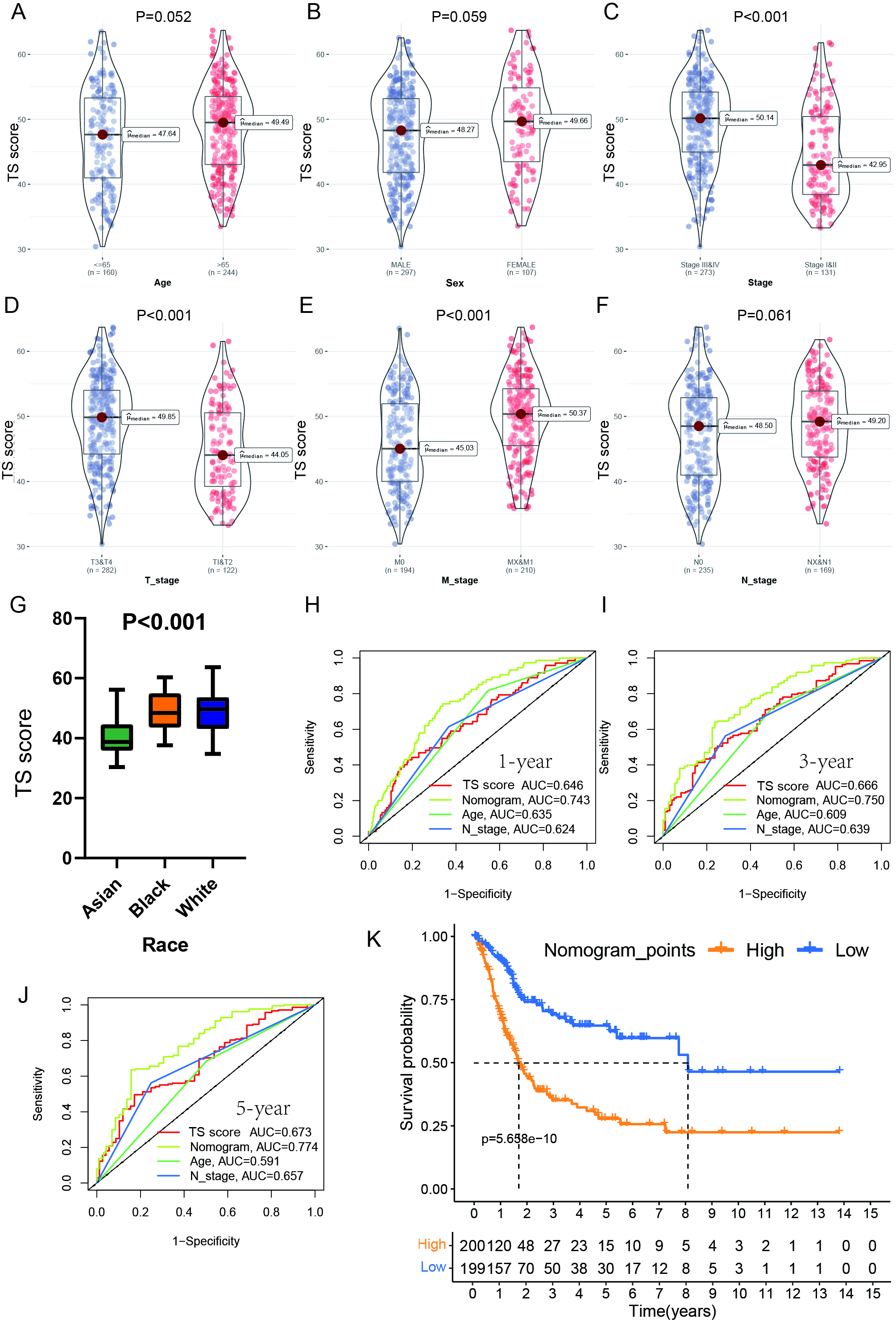

Supplement: Supplementary file 6 — Additional file 6: Figure 6. The differential analysis of age (A), sex (B), pathological stage (C), T stage (D), M stage (E), N stage (F), race (G) between high and low TS score groups in TCGA BLCA cohort. Wilcox test: p (age) = 0.052, p (sex) = 0.059, p (pathological stage) < 0.001, p (T stage) < 0.001, p (M stage) < 0.001, p (N stage) = 0.061 and p (race) < 0.001. The ROC analysis of nomogram in TCGA BLCA cohort. AUC = 0.743 (H), 0.750 (I), and 0.774 (J) at 1, 3, and 5 year. (K) Kaplan–Meier curves for high and low nomogram points in TCGA BLCA cohort. Log-rank test, p < 0.001. [file 13287_2023_3239_MOESM6_ESM.tif]

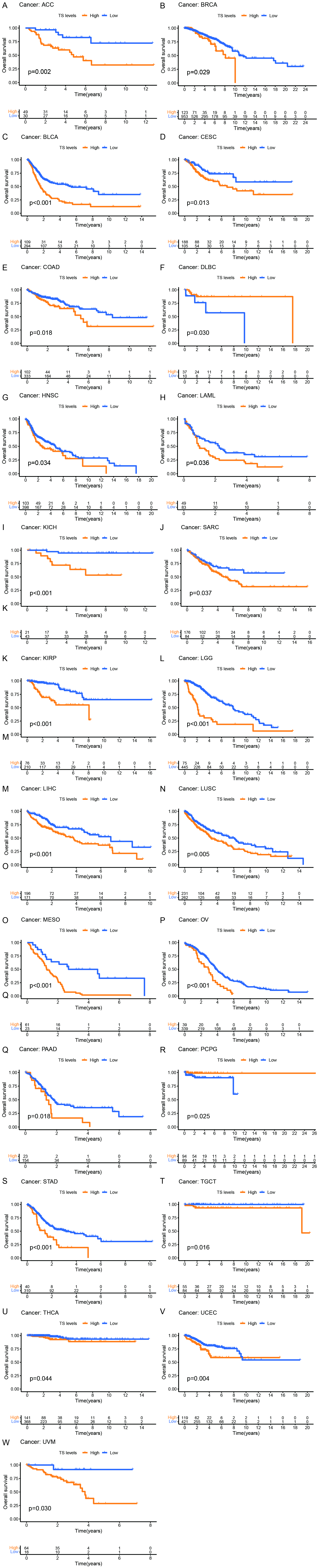

Supplement: Supplementary file 7 — Additional file 7: Figure 7. Kaplan–Meier curves of OS for high and low TS score patients in 23 types of cancer. Log-rank test, p < 0.05. [file 13287_2023_3239_MOESM7_ESM.tif]

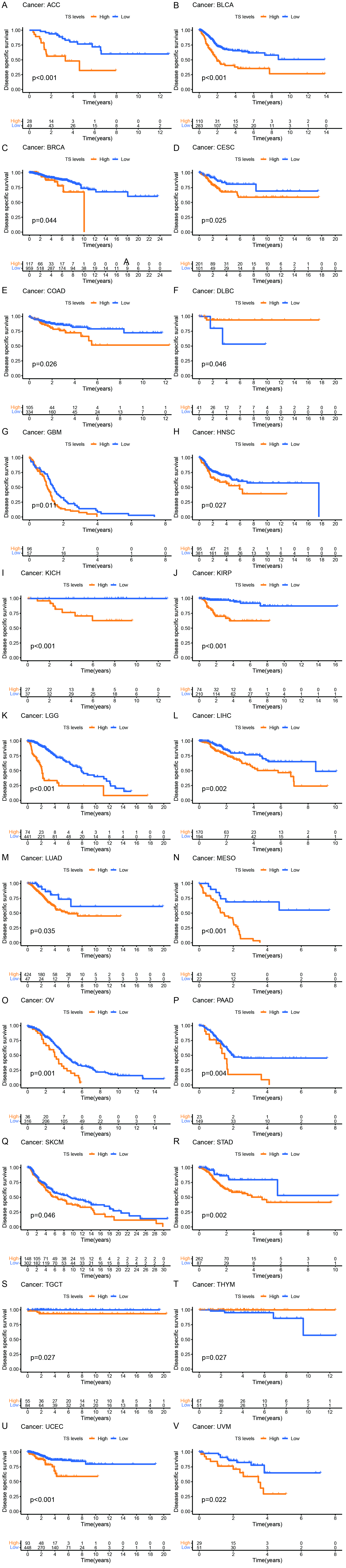

Supplement: Supplementary file 8 — Additional file 8: Figure 8. Kaplan–Meier curves of PFS for high and low TS score patients in 22 types of cancer. Log-rank test, p < 0.05. [file 13287_2023_3239_MOESM8_ESM.tif]

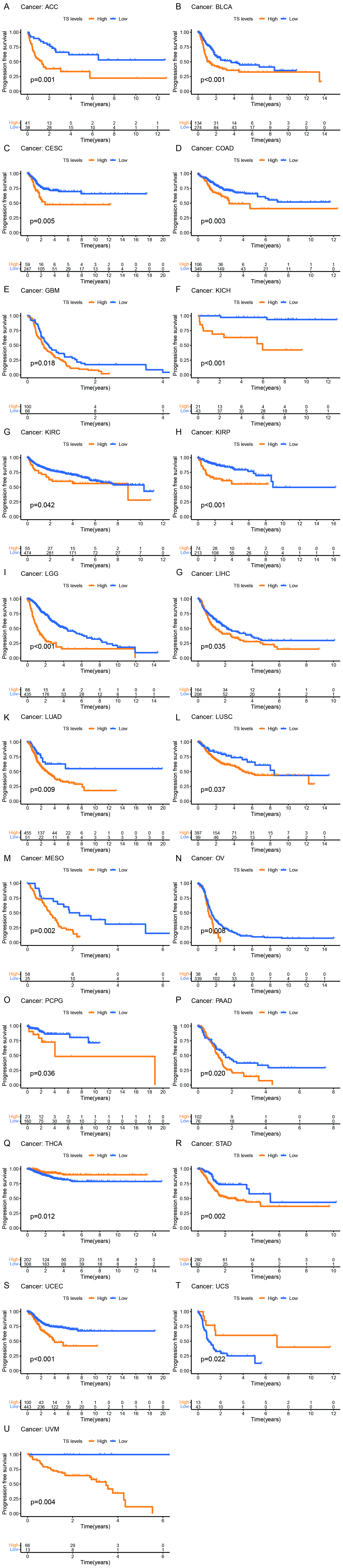

Supplement: Supplementary file 9 — Additional file 9: Figure 9. Kaplan–Meier curves of DSS for high and low TS score patients in 21 types of cancer. Log-rank test, p < 0.05. [file 13287_2023_3239_MOESM9_ESM.tif]

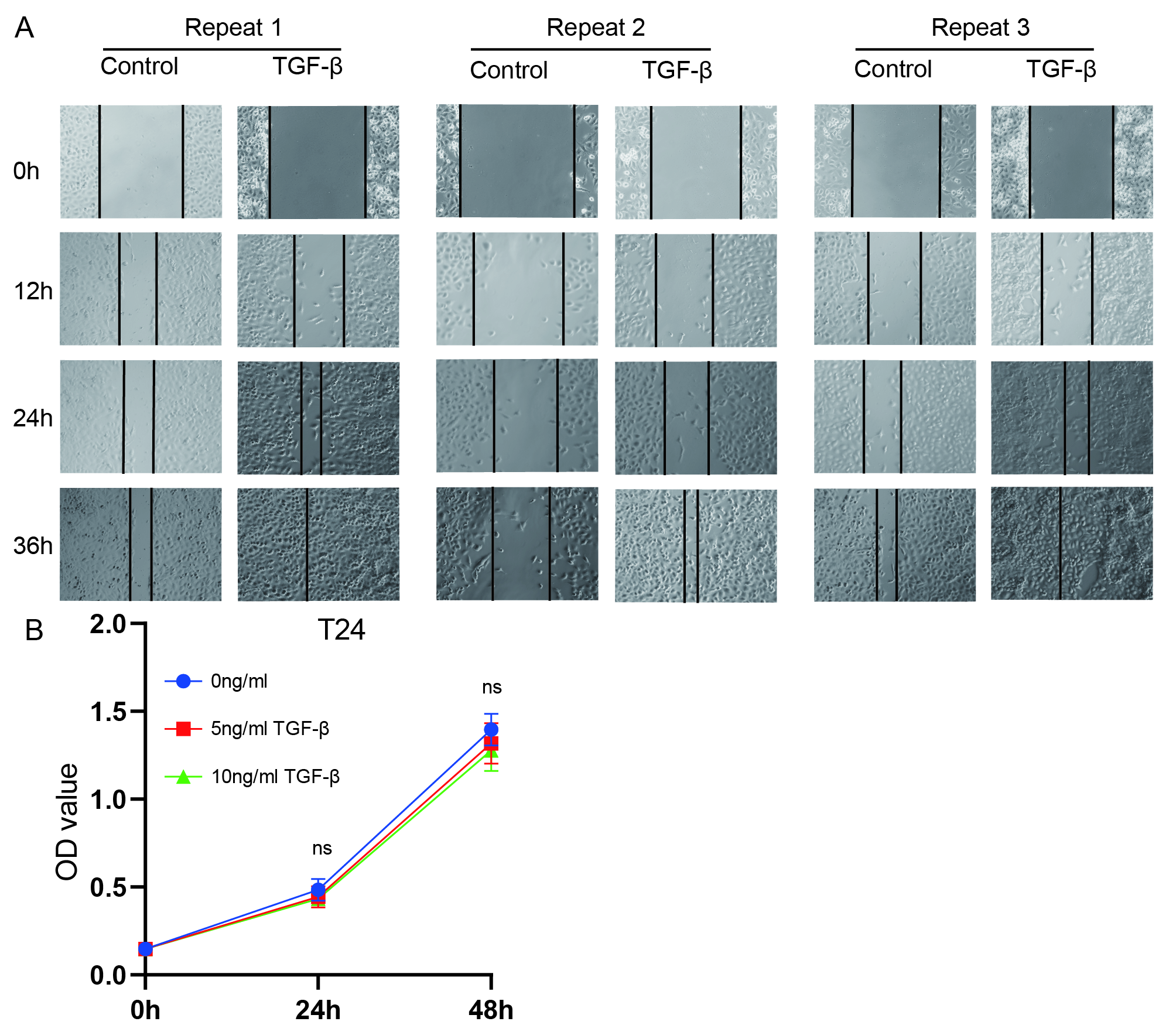

Supplement: Supplementary file 10 — Additional file 10: Figure 10. The wound filling assay (A), and cell viability (CCK-8) assay of T24 with or without TGF-β treatment (B). ANOVA analysis, p > 0.05. [file 13287_2023_3239_MOESM10_ESM.tif]

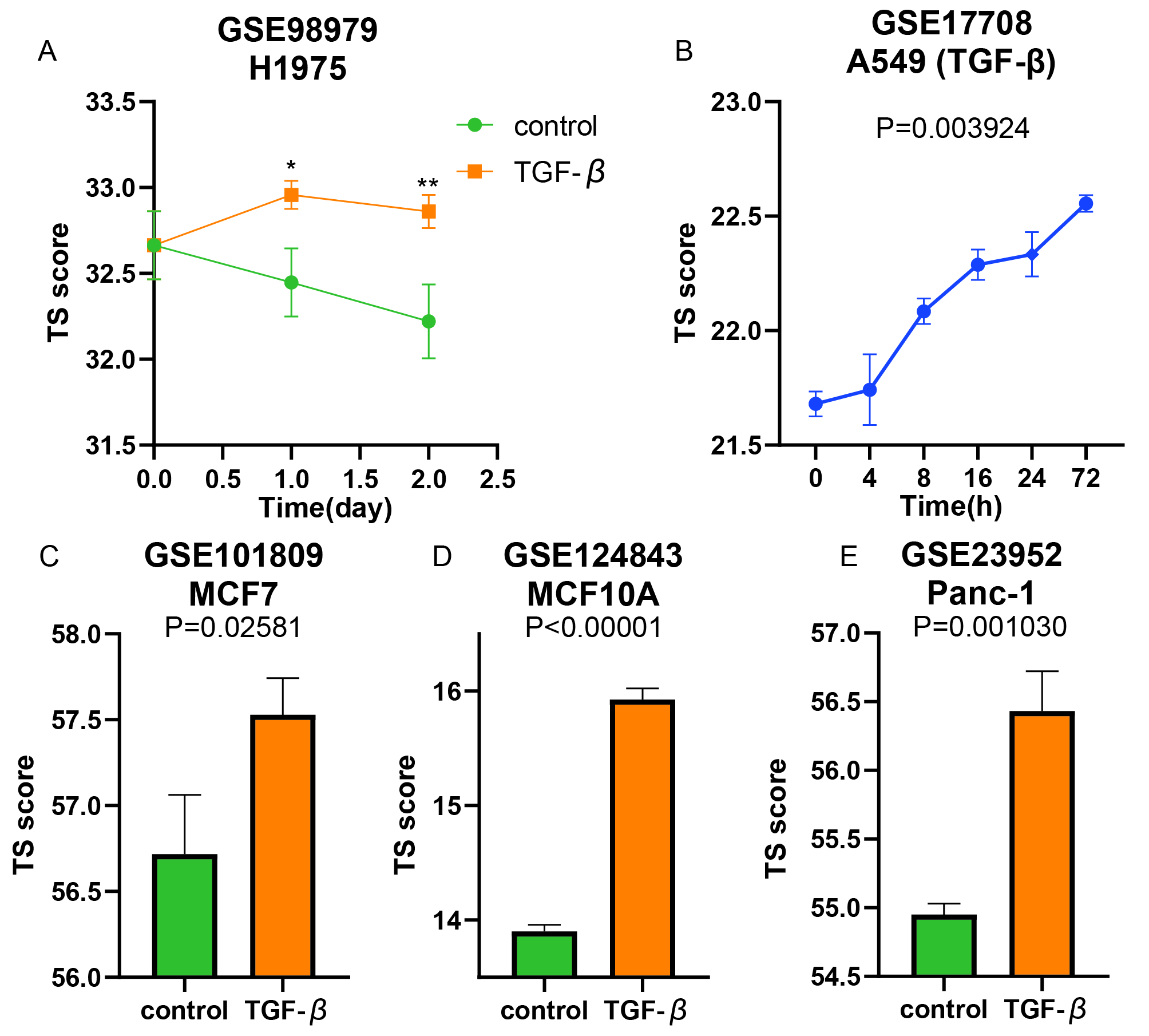

Supplement: Supplementary file 11 — Additional file 11: Figure 11. The variation of TS score of cell lines after TGF-β treatment. (A) The comparison of TS score of H1975 with or without TGF-β treatment after 1 and 2 days. Student’s t test, *p < 0.05, **p < 0.01, ***p < 0.001. (B) The comparison of TS score of A549 after TGF-β 0, 4, 8, 16, 24, and 72 hours. ANOVA analysis, p = 0.003924. (C) The comparison of TS score of MCF7 with or without TGF-β treatment. Student’s t test, p = 0.02851 (D) The comparison of TS score of MCF10A with or without TGF-β treatment. Student’s t test, p < 0.00001 (E) The comparison of TS score of Panc-1 with or without TGF-β treatment. Student’s t test, p < 0.001030. [file 13287_2023_3239_MOESM11_ESM.tif]
