# Supplementary material for: Tumor stemness score to estimate epithelial-to-mesenchymal transition (EMT) and cancer stem cells (CSCs) characterization and to predict the prognosis and immunotherapy response in bladder urothelial carcinoma
Source: Stem Cell Res Ther. 2023 Feb 1;14:15. doi: 10.1186/s13287-023-03239-1 (PMC9890713; doi:10.1186/s13287-023-03239-1)
Supplement: Supplementary file 12 — Additional file 12: Table S1. Clinical information of patients from TCGA BLCA cohort. [file 13287_2023_3239_MOESM12_ESM.docx]

| Clinical parameters | Variable | n(total=413) | Percentages(%) |
| --- | --- | --- | --- |
| Age (years) | <=65 | 162 | 39.23% |
|  | >65 | 250 | 60.53% |
| Sex | Female | 109 | 26.39% |
|  | Male | 304 | 73.61% |
| Histological grade | High grade | 388 | 93.95% |
|  | Low grade | 21 | 5.08% |
|  | Unknow | 3 | 0.73% |
| T/M/N stage | T0 | 1 | 0.24% |
|  | T1 | 3 | 0.73% |
|  | T2 | 120 | 29.06% |
|  | T3 | 196 | 47.46% |
|  | T4 | 59 | 14.29% |
|  | TX | 1 | 0.24% |
|  | Unknow (T stage) | 32 | 7.75% |
|  | M0 | 196 | 47.46% |
|  | M1 | 11 | 2.66% |
|  | MX | 202 | 48.91% |
|  | Unknow (M stage) | 3 | 0.73% |
|  | N0 | 239 | 57.87% |
|  | N1 | 47 | 11.38% |
|  | N2 | 76 | 18.40% |
|  | N3 | 8 | 1.94% |
|  | NX | 36 | 8.72% |
|  | Unknow (N grade) | 6 | 1.45% |
| Pathological stage | Stage I | 2 | 0.48% |
|  | Stage II | 131 | 31.72% |
|  | Stage III | 141 | 34.14% |
|  | Stage IV | 136 | 32.93% |
|  | Unknow | 2 | 0.48% |
| Survival status | Dead | 159 | 38.50% |
|  | Alive | 253 | 61.26% |
