# Supplementary material for: Tumor stemness score to estimate epithelial-to-mesenchymal transition (EMT) and cancer stem cells (CSCs) characterization and to predict the prognosis and immunotherapy response in bladder urothelial carcinoma
Source: Stem Cell Res Ther. 2023 Feb 1;14:15. doi: 10.1186/s13287-023-03239-1 (PMC9890713; doi:10.1186/s13287-023-03239-1)
Supplement: Supplementary file 13 — Additional file 13: Table S2. The primer sequence used in our study. [file 13287_2023_3239_MOESM13_ESM.docx]

| Gene | Primer sequence |
| --- | --- |
| CDH1-F | ATTTTTCCCTCGACACCCGAT |
| CDH1-R | TCCCAGGCGTAGACCAAGA |
| CDH2-F | AGCCAACCTTAACTGAGGAGT |
| CDH2-R | GGCAAGTTGATTGGAGGGATG |
| OCLN_F | ACAAGCGGTTTTATCCAGAGTC |
| OCLN _R | GTCATCCACAGGCGAAGTTAAT |
| ZEB1_F | GATGATGAATGCGAGTCAGATGC |
| ZEB1_R | ACAGCAGTGTCTTGTTGTTGT |
| ZEB2_F | GGAGACGAGTCCAGCTAGTGT |
| ZEB2_R | CCACTCCACCCTCCCTTATTTC |
| VIM_F | AGTCCACTGAGTACCGGAGAC |
| VIM_R | CATTTCACGCATCTGGCGTTC |
| SNAI1_F | TCGGAAGCCTAACTACAGCGA |
| SNAI1_R | AGATGAGCATTGGCAGCGAG |
| SNAI2_F | CGAACTGGACACACATACAGTG |
| SNAI2_R | CTGAGGATCTCTGGTTGTGGT |
| MMP9_F | TGTACCGCTATGGTTACACTCG |
| MMP9_R | GGCAGGGACAGTTGCTTCT |
| FN1_F | AGGAAGCCGAGGTTTTAACTG |
| FN1_R | AGGACGCTCATAAGTGTCACC |
| NFKB1_F | AACAGAGAGGATTTCGTTTCCG |
| NFKB1_R | TTTGACCTGAGGGTAAGACTTCT |
| RELA_F | ATGTGGAGATCATTGAGCAGC |
| RELA_R | CCTGGTCCTGTGTAGCCATT |
| CTNNB1_F | AGCTTCCAGACACGCTATCAT |
| CTNNB1_R | CGGTACAACGAGCTGTTTCTAC |
